# HSFA2 Functions in the Physiological Adaptation of Undifferentiated Plant Cells to Spaceflight

**DOI:** 10.3390/ijms20020390

**Published:** 2019-01-17

**Authors:** Agata K. Zupanska, Collin LeFrois, Robert J. Ferl, Anna-Lisa Paul

**Affiliations:** 1 Horticultural Science Department, Program in Plant Molecular and Cellular Biology, University of Florida, Gainesville, FL 32611, USA; zupanska@live.com (A.K.Z.); clefrois@ufl.edu (C.L.); robferl@ufl.edu (R.J.F.); 2 Interdisciplinary Center for Biotechnology Research, University of Florida, Gainesville, FL 32610, USA

**Keywords:** spaceflight, *Arabidopsis*, plant cell culture, undifferentiated cells, microgravity, heat shock

## Abstract

Heat Shock Factor A2 (HsfA2) is part of the Heat Shock Factor (HSF) network, and plays an essential role beyond heat shock in environmental stress responses and cellular homeostatic control. *Arabidopsis thaliana* cell cultures derived from wild type (WT) ecotype Col-0 and a knockout line deficient in the gene encoding HSFA2 (HSFA2 KO) were grown aboard the International Space Station (ISS) to ascertain whether the HSF network functions in the adaptation to the novel environment of spaceflight. Microarray gene expression data were analyzed using a two-part comparative approach. First, genes differentially expressed between the two environments (spaceflight to ground) were identified within the same genotype, which represented physiological adaptation to spaceflight. Second, gene expression profiles were compared between the two genotypes (HSFA2 KO to WT) within the same environment, which defined genes uniquely required by each genotype on the ground and in spaceflight-adapted states. Results showed that the endoplasmic reticulum (ER) stress and unfolded protein response (UPR) define the HSFA2 KO cells’ physiological state irrespective of the environment, and likely resulted from a deficiency in the chaperone-mediated protein folding machinery in the mutant. Results further suggested that additional to its universal stress response role, *HsfA2* also has specific roles in the physiological adaptation to spaceflight through cell wall remodeling, signal perception and transduction, and starch biosynthesis. Disabling *HsfA2* altered the physiological state of the cells, and impacted the mechanisms induced to adapt to spaceflight, and identified *HsfA2*-dependent genes that are important to the adaption of wild type cells to spaceflight. Collectively these data indicate a non-thermal role for the HSF network in spaceflight adaptation.

## 1. Introduction

Survival in a challenging environment requires a coordinated system that enables a living organism to respond appropriately to a change. The environment of the International Space Station (ISS) presents novel challenges for all terrestrial organisms, which evolved in the unit gravity of Earth. Microgravity is the most obvious challenge of spaceflight; however, other aspects of the spaceflight environment, such as radiation from galactic cosmic rays and solar energetic particles, and vibration contribute to the complexity of this novel environment. When terrestrial organisms are transported to the ISS, they respond by adjusting metabolic processes to physiologically adapt to this new spaceflight environment. One readout of the physiological changes induced by spaceflight is in the patterns of gene expression. Plants are extremely sensitive to changes in their environment, and are particularity attuned to changes in gravity. Individual genes have been implicated as important to the altered gravity physiological adaptation through assays with deletion mutants [1,2] and with specific assays of expression [3,4,5,6,7]. Genome-wide evaluations have also provided a large-scale view of the changes elicited by disrupted terrestrial gravity environments [8,9,10,11].

Plants exhibit widespread changes in their patterns of gene expression in the absence of gravity, such as in response to the spaceflight environment [4,12,13,14,15,16,17,18,19,20,21,22,23,24]. Even unicellular plants are capable of sensing a change in the gravitational environment and there have been several spaceflight experiments that examine the transcriptomes of single plant cells, and undifferentiated plants cells, which also lack traditional gravity-sensing organs [4,19,25,26,27,28]. The wealth of information on the transcriptional changes that accompany the adjustments to spaceflight begins to suggest common strategies for adjusting to the spaceflight environment; however, the underlying question of why certain genes are necessary remains unanswered. One group of genes strongly represented in many spaceflight transcriptomes is composed of the heat shock transcription factors and proteins that comprise the Heat Shock Factor network [15,19].

Heat Shock Factors (HSFs) are transcription factors that activate the expression of genes in response to almost any environmental stress and are essential to the cellular homeostatic control mechanisms (for review, please see [29]). HSF genes are evolutionarily conserved and are represented in the genomes of almost every organism. Given the conservation of the HSF network and its general roles in stress responses, it was perhaps not surprising to see HSFs represented in the response to spaceflight in plants. Their particularly strong representation in cell cultures; however, suggested HSFs could play a unique role in the spaceflight response of undifferentiated cells [4].

One hallmark of cellular stress is the accumulation of denatured proteins. The main gene targets of HSF-induced transcriptional activation are the stress response proteins that act as molecular chaperones [29,30,31,32]. These chaperones not only protect a cell from denatured protein accumulation, but also assure proper protein folding and maturation of newly synthesized proteins indispensable in response to stress. The Heat Shock Proteins (HSPs) are the main chaperones of the folding machinery, but there are also other non-chaperone proteins assisting in efficient protein maturation [33,34,35]. For instance, a pair of enzymes, including a disulfide carrier protein (protein disulfide isomerase, PDI), and a disulfide-generating enzyme (endoplasmic oxidoreductin-1, ERO1 and protein disulfide isomerase, PDI, PDIs/ERO1) catalyze oxidative protein folding by creating disulfide bonds, thereby assisting in proper protein sorting [36,37].

Protein folding occurs within the endoplasmic reticulum (ER), which also serves as the main hub for the secretory pathways and protein delivery to their final destinations. The ER uses an extensive surveillance called the Endoplasmic Reticulum Quality Control (ERQC) system, which assures that only properly folded proteins exit the ER and maintains proper physiological homeostasis in the ER [38]. When there is an accumulation of unfolded proteins in the ER, a stress signal is emitted. Initially, a cell activates the unfolded protein response (UPR) intended to increase the ER folding capacity in an attempt to refold the aberrant proteins [39,40]. If these UPR solutions to the unfolded protein accumulation problem fail, or if the accumulation of misfolded proteins is massive, then the final UPR step—the endoplasmic reticulum associated degradation (ERAD)—is activated. The ERAD eliminates and destroys the misfolded proteins via proteasome digestion, preventing further protein aggregation. The luminal lectin OS9 in cooperation with a membrane spanning protein SEL1/HRD3 recognizes the unfolded glycosylated proteins which are then ubiquitinylated by the HRD1 E3 ligase, removed from the ER lumen, and degraded by the 26S proteasome in the cytoplasm [41,42]. Taken collectively, ER homeostasis works as a sensor for environmental stimuli, and the ER stress signal initiates a subcellular stress response system that can secure protein homeostasis during environmental changes [43,44].

The *HsfA2* gene is a member of the large family of *Hsf* genes in the HSF network and is a key regulator of the defense response via HSP chaperone transcriptional activation to several types of environmental stresses, namely extreme temperatures (high and low), hydrogen peroxide, and high light intensity [45,46,47]. The HSFA2 protein has been demonstrated itself to be the main coordinator of the UPR during heat stress [48]. The critical involvement of HSFA2 in the response to extreme environments makes it an excellent target candidate for studying the effects of spaceflight on plants and to test if plants use the same universal stress response mechanism evolved terrestrially to accommodate the novel space gravitational environment.

HSFA2 may also have an additional role in the physiological adaptation to the spaceflight environment beyond the UPR induction of the chaperone-based protein folding machinery. The genes encoding HSFs and HSPs were reported to be upregulated in spaceflight in many biological systems [26,49]. The *HsfA2* gene specifically was the highest upregulated gene in the wild type *Arabidopsis* cell cultures after 12 days in space [4,19]. Moreover, HSFA2 was shown to function in amplification of the signal in response to brassinosteroids, calcium, and auxin and was reported to be affected in *Arabidopsis* in spaceflight, and therefore has the potential for playing a role in the gravity sensing signal transduction cascade [13,20,50]. In the unicellular yeast (*Saccharomyces cerevisiae*), the HSF targets represent nearly 3% of the genome and the diversity of their functions supports a broad role for HSF in coordinating the multitude of cellular processes occurring in normal and stressful conditions [51]. Since the HSFs play a role in various cellular processes such as development, cellular lifespan, cell differentiation and proliferation, the likelihood that HSFA2 will play some role in the physiological adaptation to spaceflight increases [52,53]. 

Cell lines from wild type (WT) Col-0 and an *HsfA2* knockout (HSFA2 KO) in the same Col-0 background were launched to the International Space Station (ISS) for the Cellular Expression Logic (CEL) experiment, which was a component of the Biological Research In Cannisters 17 (BRIC17) payload. The experiments here compare samples fixed in orbit after growth in space to samples grown on the ground. Descriptions and discussions will consider not only the spaceflight adaptation experience for each genotype, but also the gene expression profiles in the ground and spaceflight environments between genotypes. It was our goal to develop a better understanding of how cells, disabled in a primary regulator of environmental stress response, react to an unfamiliar environment outside of their evolutionary experience. The results of the spaceflight experiment presented here have enhanced our understanding not only of HSFA2’s role in adjusting to novel environments, but also the broader scope of the processes involved spaceflight physiological adaptation in plant cells.

## 2. Results

**Figure 1:**
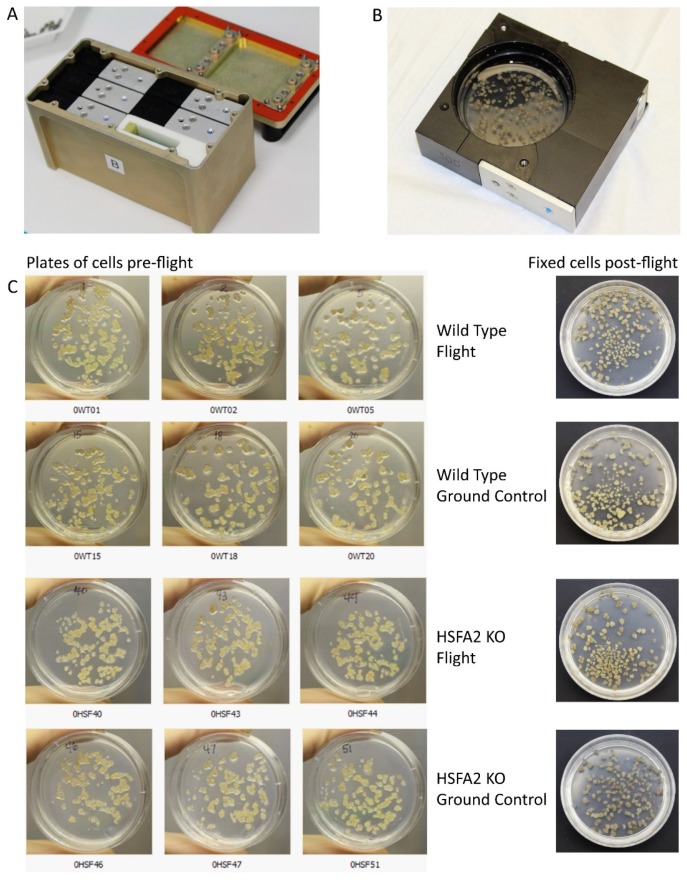
The BRIC hardware and cells flown in the BRIC17 CEL (Cellular Expression Logic) experiment. (**A**) A single BRIC (Biological Research in Canisters) hardware unit, showing five PDFUs (Petri Dish Fixation Unit) and a slot for a HOBO™ data logger; (**B**) A single PDFU containing a Petri dish of *Arabidopsis* callus cells; (**C**) Examples of replicate plates of wild type and HSFA2 KO cells from the spaceflight and ground control prior to loading into the PDFUs, along with representative photos of the fixed cells post-flight.

In this experiment, the pattern of gene expression that defined the adapted state was established after ten days of growth in the BRIC hardware in two environments: spaceflight, and ground control in the two genotypes: HSFA2 KO, and WT. Cell clusters of both genotypes were applied in comparable density for both treatments, and continued growth in the spaceflight and ground control environments (Figure 1). 

**Figure 2:**
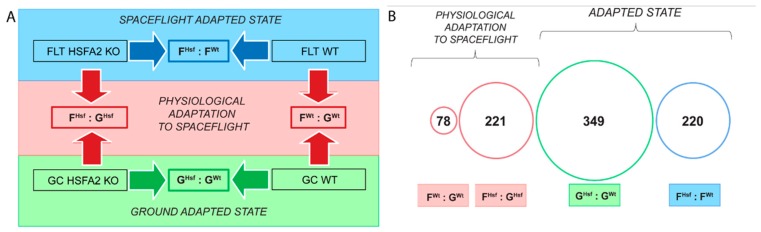
Graphical presentation of the dimensions used in the microarray data analysis and the results of thereof. (**A**) HSFA2 KO and WT mark the gene expression profiles for respective cell samples. Solid arrows represent the direction of comparison of the gene expression profiles. Red box and arrows indicate the first dimension of data analysis—gene expression profiles of spaceflight-adapted state to ground-adapted state for each of the two cell lines, thereby characterizing the physiological adaptation of each genotype to spaceflight. Green box and arrow indicate part of the second dimension of data analysis—a comparison of gene expression profiles between the two genotypes in the ground-adapted state, thereby defining the genes uniquely required by the two genotypes for ground-adapted state. Blue box and arrow indicate part of the second dimension of data analysis—a comparison of gene expression profiles between the two genotypes during spaceflight-adapted state, thereby defining the genes uniquely required by the two genotypes for spaceflight-adapted state; (**B**) A proportional graphical presentation of the significantly differentially expressed genes identified in each comparison group: 78 genes of the physiological adaptation to spaceflight in WT cells obtained from F^Wt^ : G^WT^; 221 genes of the physiological adaptation to spaceflight in HSFA2 KO cells obtained from F^Hsf^ : G^Hsf^; 349 genes of the ground-adapted state between HSFA2 KO and WT cells obtained from G^Hsf^ : G^Wt^ group; 220 genes of the spaceflight-adapted state between HSFA2 KO and WT cells obtained from F^Hsf^ : F^Wt^.

Microarray gene expression data were analyzed in two dimensions. The first or “vertical” dimension of the analysis involved the typical comparison of the gene expression profiles of the cells grown in spaceflight to those grown on the ground for each of the two cell lines (see red box in Figure 2A, and refer also to [28] for a similar experimental design). For clarity, this vertical comparison was termed the physiological adaptation to the spaceflight environment of either HSFA2 KO or WT cells. Genes identified in this vertical comparison contribute to understanding which cellular processes were sensitive to spaceflight in each genotype. The second or “horizontal” dimension of the analysis involved comparison of gene expression profiles between the two genotypes within the same environment: ground (see green box of Figure 2A) and spaceflight (see blue box of Figure 2A). In the ground horizontal comparison, gene expression in HSFA2 KO cells on the ground was compared to gene expression in WT cells on the ground, thus defining unique genes of the ground adapted state for each genotype. Similarly, in a spaceflight horizontal comparison, the gene expression in HSFA2 KO cells in spaceflight was compared to gene expression in WT cells in spaceflight, thus defining unique genes required for the spaceflight adapted state in each genotype. Finally, the sets of genes obtained from a comparison of the first and second dimension were analyzed together, and their interdependence was examined. 

The gene expression datasets were abbreviated as follows: Ground Control WT (G^Wt^), Ground Control HSFA2 KO (G^Hsf^), Spaceflight WT (F^Wt^) and Spaceflight HSFA2 KO (F^Hsf^). The WT physiological adaptation to spaceflight was identified in the F^Wt^ : G^Wt^ comparison group (see red box on the left in Figure 2A), the HSFA2 KO physiological adaptation to spaceflight was identified in the F^Hsf^ : G^Hsf^ comparison group (red box on the right in Figure 2A), the genotypic adaptation to ground was identified in the G^Hsf^ : G^Wt^ comparison group (see green box at the bottom in Figure 2A), and the genotypic adaptation to spaceflight was identified in the F^Hsf^ : F^Wt^ comparison group (blue box on top in Figure 2A). The genes that were differentially expressed in these comparisons are outlined in the following sections in terms of the numbers of differentially expressed genes represented in general ontology categories. Full annotations of the genes in each category are presented in Appendix A Table A1 and Table A2.

### 2.1. The HsfA2 Expression Levels across Samples

The raw gene expression values in microarrays, measured as a fluorescence signal intensity, showed that the *HsfA2* transcript was abundant in the WT undifferentiated cells in the ground control samples. The average raw expression of the *HsfA2* gene in the 4 biological replicates of the WT ground control cells was 4171 (see Figure S1 in the Supplementary Material). In contrast, the average raw *HsfA2* gene expression in the 3 biological replicates of the HSFA2 KO ground control cells was only 39. Thus, there was a deficit of −7.27 log_2_ Fold Change (*P*-value 2.9 × 10^−9^) of the *HsfA2* transcript in HSFA2 KO cells than in WT cells on the ground, demonstrating that the T-DNA insertion mutation severely depressed *HsfA2* expression. 

There was no statistically significant difference in the *HsfA2* expression levels between spaceflight and corresponding ground control for either the WT cells or the HSFA2 KO cells (see Figure S1 in the Supplementary Material). 

### 2.2. Different Genes Characterize the Physiological Adaptation of the WT and HSFA2 KO Cells to Spaceflight

#### 2.2.1. The Genes Characterizing the Physiological Adaptation of WT Cells to Spaceflight—F^Wt^ : G^Wt^

**Figure 3:**
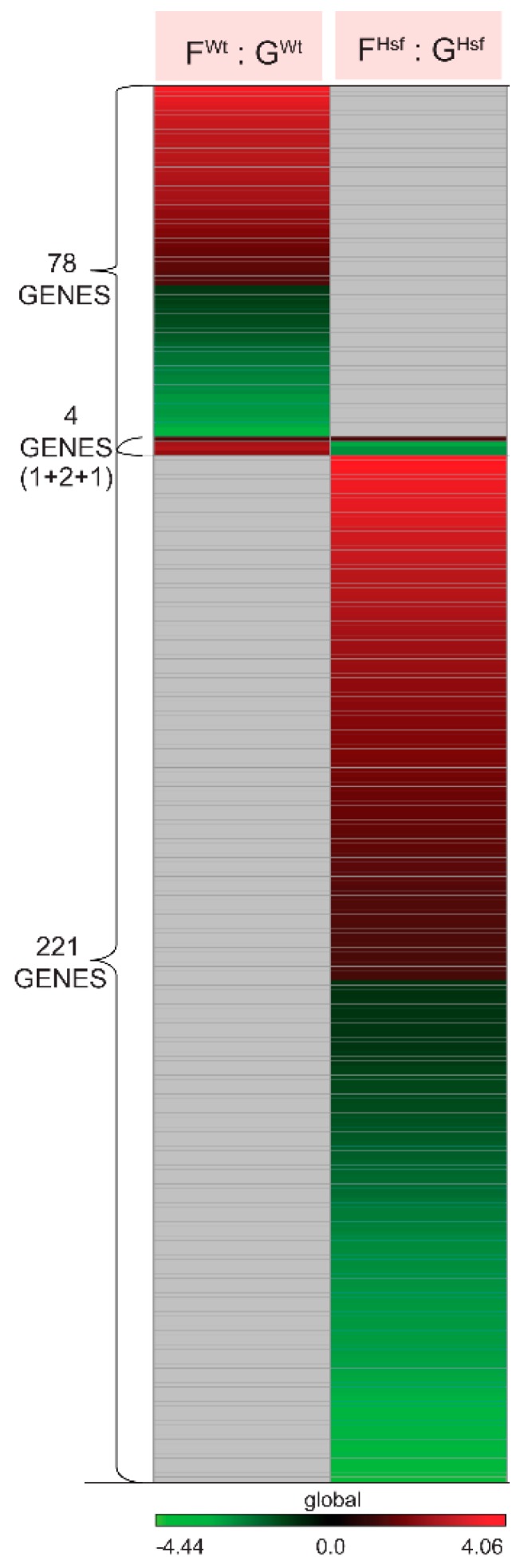
Heat map visualizing the expression of significantly differentially expressed genes: 78 genes of the physiological adaptation to spaceflight in WT cells obtained from the F^Wt^ : G^Wt^ group comparison, and 221 genes of the physiological adaptation to spaceflight in HSFA2 KO cells obtained from the F^Hsf^ : G^Hsf^ group comparison.

The genes involved in physiological adaptation to spaceflight in WT cells were identified by comparing the gene expression profiles in WT spaceflight cells (F^Wt^) to the WT ground control cells (G^Wt^) in the F^Wt^ : G^Wt^ group comparison (see Figure 2A). There were 78 genes significantly differentially expressed between spaceflight and ground control at *P*-value < 0.01 and log_2_ Fold Change > 1; 46 genes were upregulated and 32 genes were downregulated (see Figure 2B, Figure 3, File S1 GO 78 FWtGWt in the Supplementary Material). The microarray data are publicly available from GEO (GSE95388) and GeneLab (number TBD).

The upregulated genes identified from the F^Wt^ : G^Wt^ group comparison represented genes that were overexpressed in WT cells in spaceflight compared to the WT cells on the ground. These genes primarily fell under two GO terms of biological process ontology: response to stimulus (GO:0050896) and regulation of biological processes (GO:0050789). Many of these genes are associated with defense, wounding, and cell wall metabolism (Table A1). 

The downregulated genes identified from F^Wt^ : G^Wt^ group comparison represented genes that were downregulated in WT cells in spaceflight compared to the WT cells on the ground. These genes primarily fell under the regulation of biological process GO term (GO:0050789) and under the categories of endomembrane systems (GO:0012505) and Golgi associated (GO:0005794). Many of these genes are associated with cellular transport and receptors (Table A1).

#### 2.2.2. HSFA2 KO Cells Changed the Expression of Three Times More Genes than Seen in WT Cells—F^Hsf^ : G^Hsf^

The genes involved in physiological adaptation to spaceflight in HSFA2 KO cells were identified by comparing the gene expression profiles in HSFA2 KO spaceflight cells (F^Hsf^) to HSFA2 KO ground control cells (G^Hsf^) in the F^Hsf^ : G^Hsf^ group comparison (see Figure 2A). There were 221 genes significantly differentially expressed between spaceflight and ground control at *P*-value < 0.01 and log_2_ Fold Change > 1; 112 genes were upregulated, and 109 genes were downregulated (see Figure 2B, Figure 3, File S2 GO 221 FHsfGHsf in the Supplementary Material).

The upregulated genes identified from F^Hsf^ : G^Hsf^ group comparison represented genes that were overexpressed in HSFA2 KO cells in spaceflight compared to the HSFA2 KO cells on the ground. These genes primarily fell under 3 GO terms of biological process ontology: the defense response genes to other organism (GO:0098542), including response to wounding, cellular response to sucrose starvation (GO:0043617) and valine, leucine, and isoleucine degradation (GO:0009083) (see Table A1).

The downregulated genes identified from F^Hsf^ : G^Hsf^ group comparison represented genes that were underexpressed in HSFA2 KO cells in spaceflight compared to the HSFA2 KO cells on the ground. These genes primarily fell under 2 GO terms of biological process ontology: closely related growth, developmental process (GO:0032502) and anatomical structure morphogenesis (GO:0009653), particularly those involved in cell elongation, cell wall loosening, and multidimensional cell growth. These downregulated genes also fell under 2 GO terms of the cellular component ontology: plant-type cell wall (GO:0009505) and plasma membrane (GO:0005886) (see Table A1).

### 2.3. WT and HSFA2 KO Show More Difference in Gene Expression Profiles in Their Ground-Adapted State than in Spaceflight-Adapted State

#### 2.3.1. Genotype Specific Genes of the Ground-Adapted State—G^Hsf^ : G^Wt^

**Figure 4:**
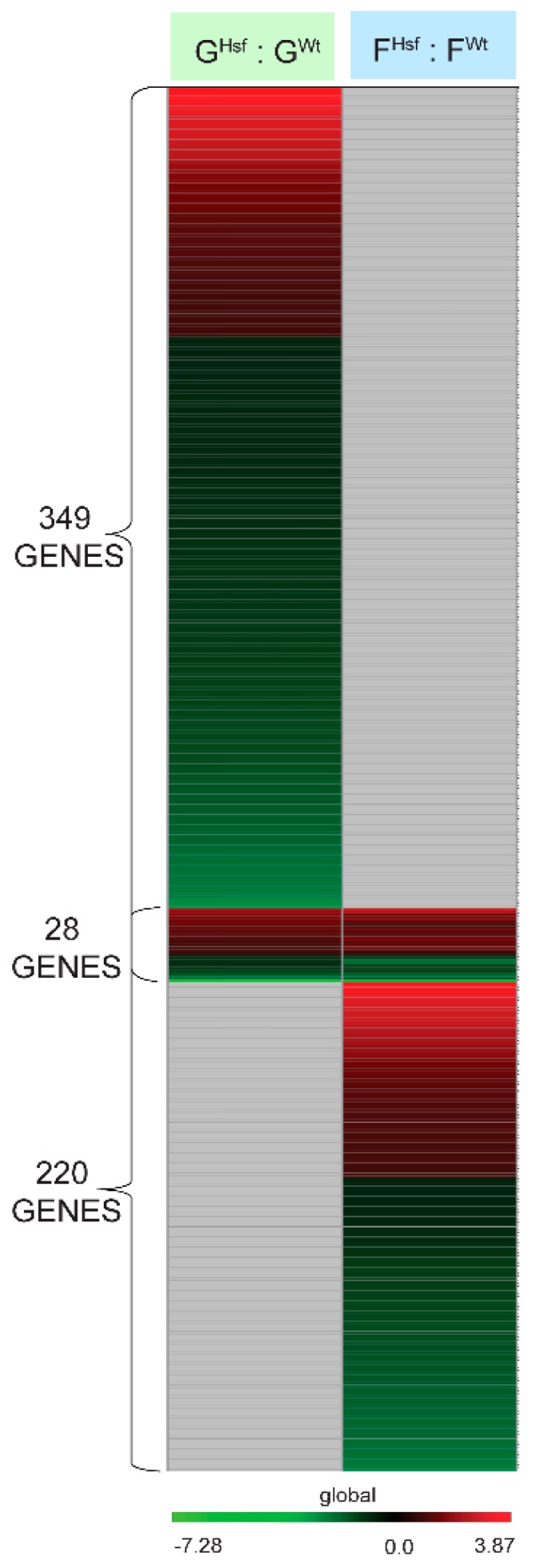
Heat map visualizing the expression of significantly differentially expressed genes: 349 genes of the ground-adapted state between HSFA2 KO and WT cells obtained from G^Hsf^ : G^Wt^ group comparison and 220 genes of the spaceflight-adapted state between HSFA2 KO and WT cells obtained from F^Hsf^ : F^Wt^ group comparison.

Comparison of the gene expression profiles of the ground-adapted states between the HSFA2 KO and WT cells shows the consequences of the *HsfA2* gene loss for cells in the ground environment. The genes differentially expressed in the ground-adapted state between HSFA2 KO cells and WT cells were identified by comparing the gene expression profiles in HSFA2 KO ground cells (G^Hsf^) to WT ground control cells (G^Wt^) in the G^Hsf^ : G^Wt^ group comparison (see Figure 2). There were 349 genes significantly differentially expressed between WT and HSFA2 KO cells in the ground-adapted state at *P*-value < 0.01 and log_2_ Fold Change > 1; 115 genes were upregulated and 234 genes were downregulated (see Figure 2B, Figure 4, File S3 GO 349 GHsfGWt in the Supplementary Material). Genotypes in ground-adapted state displayed the greatest differences in gene expression profiles than in any other comparison group. Thirty-three percent of genes had an enhanced expression in the ground-adapted state in HSFA2 KO cells compared to WT cells, and 67% had diminished expression (see Figure S2 in the Supplementary Material).

In the HSFA2 KO and WT ground-adapted state, the upregulated genes identified from the G^Hsf^ : G^Wt^ group comparison represented genes that were overexpressed in cells disabled in HSFA2 KO on the ground compared to WT cells on the ground. These genes primarily fell under multiple GO terms of biological process ontology: response to stimulus (GO:0050896) and defense response to other organism (GO:0098542), particularly to: nematode, fungus, bacterium and to wounding, water deprivation (GO:0009414) related to high salinity, response to endoplasmic reticulum stress (ER stress) (GO:0034976) and unfolded protein response (UPR) (GO:0006986) and response to misfolded protein (GO:0051788), localization (GO:0051179), transport (GO:0006810), establishment of localization (GO:0051234) (see Table A2). 

In the HSFA2 KO and WT ground-adapted state, the downregulated genes identified from the G^Hsf^ : G^Wt^ group comparison represented genes that were underexpressed in cells disabled in HSFA2 KO on the ground compared to WT cells on the ground. These genes primarily fell under multiple GO terms of biological process ontology: response to stimulus (GO:0050896) and response to stress (GO:0006950), including chaperones and non-chaperones, response to oxidative stress (GO:0006979), response to reactive oxygen species (GO:0000302), response to hydrogen peroxide (GO:0042542), response to sucrose stimulus (GO:0009744) and cellular amino acid and derivative metabolic process (GO:0006519), such as biosynthesis of phenylalanine, asparagine, tryptophan and flavonoid (see Table A2). 

#### 2.3.2. Genotype Specific Genes of the Spaceflight-Adapted State—F^Hsf^ : F^Wt^

A comparison of the gene expression profiles of spaceflight-adapted states between the HSFA2 KO and WT cells shows cells’ adaptation to spaceflight environment if the HSFA2 function was disabled. Thus, these genes indicate the genotypic adaptation to spaceflight. The genes differentially expressed in the spaceflight-adapted state between HSFA2 KO cells and WT cells were identified by comparing the gene expression profiles in HSFA2 KO spaceflight cells (F^Hsf^) to WT spaceflight cells (F^Wt^) in the F^Hsf^ : F^Wt^ group comparison (see Figure 2A). There were 220 genes significantly differentially expressed between HSFA2 KO and WT cell samples in spaceflight (see Figure 2B, Figure 4, File S4 GO 220 FHsfFWt in the Supplementary Material). The 95 genes were more highly expressed in HSFA2 KO cells than in WT cells in spaceflight, while 125 genes were downregulated in HSFA2 KO cells as compared to WT cells in spaceflight. In the HSFA2 KO and WT spaceflight-adapted state, the upregulated genes identified from the F^Hsf^ : F^Wt^ group comparison, represented genes that were overexpressed in cells disabled in *HsfA2* in spaceflight compared to WT cells in spaceflight. These genes primarily fell under multiple GO terms of biological process ontology: defense response to other organism (GO:0098542), response to topologically incorrect protein (GO:0035966), response to unfolded protein (UPR) (GO:0006986) and endoplasmic reticulum-associated degradation (ERAD) (GO:0036503), as well as protein localization (GO:0008104), establishment of protein localization (GO:0045184), protein transport (GO:0015031), vesicle-mediated transport (GO:0016192), transport (GO:0006810), establishment of localization (GO:0051234), including protein processing in endoplasmic reticulum, ER to Golgi vesicle-mediated transport and secondary metabolic process (GO:0019748) (see Table A2). Additionally, these upregulated genes represented plasma membrane GO term (GO:0005886) including plasma membrane receptor kinases of the cellular component ontology (see Table A2).

In the HSFA2 KO and WT spaceflight-adapted state, the downregulated genes identified from the F^Hsf^ : F^Wt^ group comparison represented genes that were underexpressed in cells disabled in *HsfA2* in spaceflight compared to WT cells in spaceflight. These genes primarily fell under 3 GO terms of cellular compartment ontology: cell wall (GO:0005618), external encapsulating structure (GO:0030312) including xylem development, cell wall macromolecule metabolic process, plastid (GO:0009536) and energy reserve metabolic process (GO:0006112) including starch biosynthetic process (GO:0019252) (see Table A2).

### 2.4. Occurrence of Individual Genes and Gene Function Across Collective Comparisons of Physiological Adaptation, Ground and Spaceflight-Adapted States

#### 2.4.1. In the Response to Spaceflight, There Were Only Four Differentially Expressed Genes Represented in Both the WT and HSFA2 KO Comparisons (F^Wt^ : G^Wt^ to F^Hsf^ : G^Hsf^)

**Figure 5:**
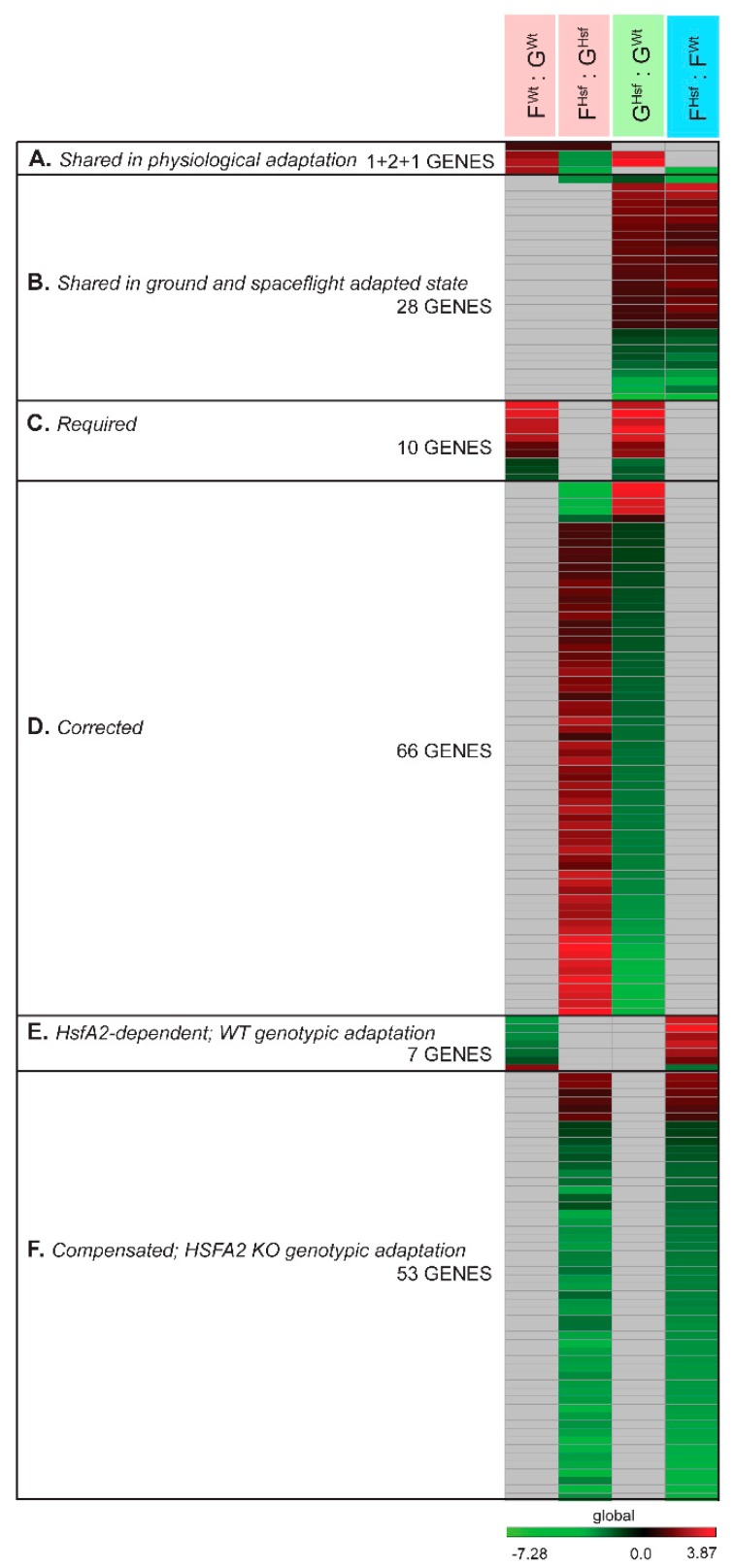
The heat map visualization of the selected significantly differentially expressed genes arranged by a specific expression patterns in the four comparison groups F^Wt^ : G^Wt^, F^Hsf^ : G^Hsf^, G^Hsf^ : G^Wt^, F^Hsf^ : F^Wt^. A through F designate a category for respective expression pattern. (**A**) genes were selected by presence among the physiological adaptation genes: 4 genes shared by the 78 of the physiological adaptation to spaceflight in WT cells obtained from F^Wt^ : G^Wt^ group comparison and 221 genes of the physiological adaptation to spaceflight in HSFA2 KO cells obtained from F^Hsf^ : G^Hsf^ group comparison; (**B**) genes were selected by presence among the ground and spaceflight-adapted state genes: 28 genes that showed the same differential expression pattern in the ground-adapted state between HSFA2 KO and WT cells obtained from G^Hsf^ : G^Wt^ group and in the spaceflight-adapted state between HSFA2 KO and WT cells obtained from F^Hsf^ : F^Wt^; (**C**) genes were selected by presence among the WT physiological adaptation and ground-adapted state genes: 10 genes that showed the same differential expression pattern in the physiological adaptation to spaceflight in WT cells obtained from F^Wt^ : G^Wt^ comparison group and the ground-adapted state between HSFA2 KO and WT cells obtained from G^Hsf^ : G^Wt^ group. These genes were categorized as Required; (**D**) Genes were selected by presence among the HSFA2 KO physiological adaptation and ground-adapted state genes: 66 genes that showed opposite differential expression pattern in the physiological adaptation to spaceflight in HSFA2 KO cells obtained from F^Hsf^ : G^Hsf^ comparison group and the ground-adapted state between HSFA2 KO and WT cells obtained from G^Hsf^ : G^Wt^ group. These genes were categorized as Corrected; (**E**) Genes were selected by presence among the WT physiological adaptation and spaceflight-adapted state genes: 7 genes that showed the opposite differential expression pattern in the physiological adaptation to spaceflight in WT cells obtained from F^Wt^ : G^Wt^ comparison group and the spaceflight-adapted state between HSFA2 KO and WT cells obtained from F^Hsf^ : F^Wt^ group. These genes were categorized as *HsfA2*-dependent. In addition, reflect WT genotypic adaptation; (**F**) genes were selected by presence among the HSFA2 KO physiological adaptation and spaceflight-adapted state genes: 53 genes that showed the same differential expression pattern in the physiological adaptation to spaceflight in HSFA2 KO cells obtained from F^Hsf^ : G^Hsf^ comparison group and the spaceflight-adapted state between HSFA2 KO and WT cells obtained from F^Hsf^ : F^Wt^ group. These genes were categorized as Compensated, and reflect HSFA2 KO genotypic adaptation.

When the 78 differentially expressed genes in the F^Wt^ : G^Wt^ group comparison were compared to the 221 genes differentially expressed in the F^Hsf^ : G^Hsf^ group comparison, only one gene changed in the exact same way, At2g03760, which encodes a putative steroid sulfotransferase (see Figure 3, Figure 5A, File S5 Gene lists in the Supplementary Material). At2g03760 was upregulated in spaceflight cells relative to their ground counterparts in both cell lines. 

Two of the genes differentially expressed in spaceflight for both HSFA2 KO and WT cells were expressed in opposite directions. During spaceflight, these genes were upregulated in WT cells, while being down regulated HSFA2 KO cells (see Figure 5A, File S5 Gene lists in the Supplementary Material). These genes were: At3g10400-RNA recognition motif, and CCHC-type zinc finger domain containing protein and At4g03570-Cystatin/monellin superfamily protein. However, these two genes showed significant differential expressions in ground-adapted state between HSFA2 KO and WT cells, being overexpressed in HSFA2 KO cells relative to WT cells. The genes were not differentially expressed in the spaceflight-adapted state between HSFA2 KO and WT cells. Therefore, although the genes seemed to be engaged conversely in the physiological adaptation in two cell lines, their expression adjustments in either HSFA2 KO or WT cells resulted in no different expression level in spaceflight-adapted state. The differential expression level on the ground caused HSFA2 KO cells to diminish the expression of these genes, and WT cells to increase in the physiological adaptation to spaceflight. 

One additional gene was expressed in opposite directions in response to spaceflight. At4g02460-PMS1 DNA mismatch repair protein was upregulated in WT and downregulated in HSFA2 KO cells (see Figure 5A, File S5 Gene lists in the Supplementary Material).

#### 2.4.2. Only 28 Genes Differentially Expressed Between the Two Genotypes in the Ground-Adapted State were also Differentially Expressed in the Spaceflight-Adapted State (G^Hsf^ : G^Wt^ to F^Hsf^ : F^Wt^)

When the 349 genes differentially expressed in the G^Hsf^ : G^Wt^ group comparison were juxtaposed to the 220 genes of differentially expressed in the F^Hsf^ : F^Wt^ group comparison, only 28 genes showed the same differential expression (see Figure 4, Figure 5B). 

Among these genes were those representing response to toxic substance ontology (At2g29490-ATGSTU1 glutathione S-transferase tau 1, At2g29470-ATGSTU3 glutathione S-transferase tau 3, At1g17170-ATGSTU24 glutathione S-transferase tau 24), and transport, establishment of localization, and localization (At3g44340-CEF ER to Golgi vesicle-mediated transport, At1g29310-SecY protein transport family protein, At1g78570-RHM1 RHAMNOSE BIOSYNTHESIS 1 dTDP-glucose 4,6-dehydratase, At1g71140-MATE efflux family protein, At5g54860-Major facilitator superfamily protein).

#### 2.4.3. Required and Corrected Genes for the Spaceflight-Adapted State

Some genes differentially expressed in the ground-adapted state between WT and HSFA2 KO were involved in the physiological adaptation to spaceflight in WT or HSFA2 KO cells. The differential expression of the 349 genes expressed in the ground-adapted state, the G^Hsf^ : G^Wt^ group comparison, was assessed in the WT physiological adaptation to spaceflight, the F^Wt^ : G^Wt^ comparison group, and in the HSFA2 KO physiological adaptation to spaceflight, the F^Hsf^ : G^Hsf^ comparison group. 

If the gene differentially expressed between the ground-adapted states in genotypes participated in the physiological adaptation of the WT cells but not of HSFA2 KO cells, then it was defined as required. For a gene to be required it had to meet these criteria:

- WT cells changed the expression of the gene in spaceflight compared to ground control.
- WT cells differentially expressed the gene on the ground compared to HSFA2 KO cells on the ground.
- HSFA2 KO cells did not change the gene’s expression in spaceflight compared to ground control.
- However, the level of expression of the gene in WT spaceflight was similar to the expression of the gene in HSFA2 KO in spaceflight.

Therefore, required genes are those whose expression levels in WT cells in spaceflight match the expression in HSFA2 KO in spaceflight. HSFA2 KO cells already have the space-adapted expression level. There were 10 genes that met these criteria (see Figure 5C, File S5 Gene lists in the Supplementary Material). 

If the gene differentially expressed between the ground-adapted states in genotypes participated in the physiological adaptation of the HSFA2 KO cells but not of the WT cells, then it was defined as corrected. For a gene to be classified as corrected it needed to meet these criteria:

- HSFA2 KO cells changed the expression of the gene in spaceflight compared to ground control.
- HSFA2 KO cells differentially expressed the corrected gene on the ground compared to WT cells on the ground.
- WT cells did not change the corrected gene expression in spaceflight compared to ground control.
- However, the level of expression of the gene in HSFA2 KO in spaceflight is similar to the expression level of the gene in WT in spaceflight.

Therefore, corrected genes are those whose expression levels in HSFA2 KO cells in spaceflight match the WT expression levels in spaceflight. These are genes that HSFA2 KO differentially expressed to match WT levels in order to adapt to spaceflight. These genes would not be revealed in an examination of WT cells alone. There were 66 genes that met these criteria (see Figure 5D, File S5 Gene lists in the Supplementary Material). Thus, these 66 genes were considered to be corrected in the HSFA2 KO physiological adaptation to spaceflight in that their expression levels were returned to WT levels in order adapt to spaceflight. Only 5 genes out of 66 were overexpressed in HSFA2 KO cells on the ground relative to WT cell on the ground and thus were downregulated in the HSFA2 KO physiological adaptation to spaceflight to even the WT expression level in the spaceflight-adapted state. The 61 genes showed diminished expression on the ground in HSFA2 KO cells relative to WT cells on the ground, but were upregulated during spaceflight to match the WT expression level in the spaceflight-adapted state. These 5 genes included: At3g44800-Meprin and TRAF (MATH) homology domain-containing protein of unknown function, At5g52260-myb19 a transcription factor, At2g35340-MEE29 maternal effect embryo arrest 29, At2g46960-CYP709B1 cytochrome P450 functioning in oxidation-reduction processes, and At5g15350-ENODL17 early nodulin-like protein 17 associated with cell wall pectin metabolic process. The 61 genes included genes associated with: response to wounding, mechanical stress, cell wall, valine, leucine and isoleucine degradation, as well as transmembrane transporter and nitrate transporters genes (see Appendix A Table A3).

#### 2.4.4. Genotypic-Specific Spaceflight-Adapted State

A comparison of the spaceflight gene expression patterns between the two genotypes revealed 220 differentially expressed genes between WT and HSFA2 KO. The expression of these 220 differentially expressed in the spaceflight-adapted state (F^Hsf^ : F^Wt^) was compared to the WT physiological adaptation to spaceflight, the F^Wt^ : G^Wt^ comparison group, and the HSFA2 KO physiological adaptation to spaceflight, the F^Hsf^ : G^Hsf^ comparison group. 

##### *HsfA2*-Dependent Genes in the WT Genotypic Spaceflight Adaptation

In the vertical comparison between spaceflight and ground gene expression patterns, there were 7 genes differentially expressed in WT that were not differentially expressed in HSFA2 KO cells. These genes were not differentially expressed between WT and HSFA2 KO in the ground-adapted state, but were differentially expressed between genotypes in the spaceflight-adapted state (see Figure 5E, File S5 Gene lists in the Supplementary Material). Thus, these genes were *HsfA2*-dependent, as they were adapted to the spaceflight level only if the *HsfA2* gene was functional. These genes represent the WT genotypic adaptation to spaceflight. 

All genes but one (At4g07960-ATCSLC12 Cellulose-synthase-like C12 localized to Golgi apparatus, plasmodesma), were downregulated in the WT physiological adaptation and resulted in the higher expression in spaceflight-adapted state in HSFA2 KO than in WT cells. These 6 genes were: At5g19070-SNARE associated Golgi protein family involved in proline transport and localized to plasma membrane, At1g78980-SRF5 STRUBBELIG-receptor family 5 functioning in transmembrane receptor protein tyrosine kinase signaling pathway and localized to extracellular region, two genes, At1g66640-RNI-like superfamily protein and At2g36560-AT hook motif DNA-binding family protein, localized to nucleus, and At2g43240-Nucleotide-sugar transporter and At1g32830-transposable element gene (see Table A3). 

##### Compensated Genes for Disabled *HsfA2* in the HSFA2 KO Spaceflight Adaptation

In the vertical comparison of gene expression patterns between spaceflight and ground controls for WT and HSFA2 KO cells, there were 53 genes differentially expressed in HSFA2 KO that were not differentially expressed in WT. As with the *HsfA2*-dependent genes described above, these genes were not differentially expressed between the two genotypes in the ground-adapted state, but were differentially expressed between genotypes in the spaceflight-adapted state (see Figure 5F, File S5 Gene lists in the Supplementary Material). These 53 genes were differentially expressed in the spaceflight-adapted state because HSFA2 KO cells engaged these genes in the physiological adaptation to spaceflight, while the WT cells did not. Thus, these genes compensated for the lack of functional *HsfA2* gene. These genes represent the HSFA2 KO genotypic adaptation to spaceflight. 

There were 6 genes out of the 53 which were upregulated in the physiological adaptation to spaceflight in HSFA2 KO cells which resulted in the higher expression level in spaceflight-adapted state in HSFA2 KO cells than in WT cells. These genes were associated with ER stress, UPR and vesicle-mediated transport (see Table A3). The remaining 49 genes were all downregulated in the HSFA2 KO physiological adaptation and exhibited diminished expression level in the spaceflight-adapted state in spaceflight in HSFA2 KO relative to WT cells. These genes included genes associated with intra-Golgi vesicle-mediated transport and calcium pump (see Table A3).

## 3. Discussion

Undifferentiated *Arabidopsis thaliana* cell cultures were flown to the ISS as part of the BRIC17 CEL experiment to enhance the understanding of how cells lacking HSFA2, a key transcription factor involved in the response to terrestrial stress stimuli, physiologically adapt to the novel environment of spaceflight. The WT cells and the cells disabled in expression of the *HsfA2* gene both survived their orbits in the ISS, indicating that they both had adapted their physiology to the novel environment despite their genotypic differences. Cells disabled in HSFA2 function used substantially more and substantially different gene expression profiles to achieve a spaceflight-adapted state compared to WT cells. This observation leads to a remarkable conclusion about the role of the HSF network in the entire adaptation processes to spaceflight—proficient spaceflight adaptation requires a functional HSF network.

This conclusion suggests that various aspects of HSFA2 and the HSF network illuminate important components of spaceflight adaptation in plant cells. 

### 3.1. Universal and Specific Aspects of the HSFA2 Role in the Adaptation to Spaceflight

Inevitably, the physiological adaptation to a change in the environment, including the novel change of ground to spaceflight, requires de novo protein synthesis followed by protein maturation and transport. Therefore, when a population of cells transitions to the spaceflight environment, the demands on a cell to synthesize and mature new proteins manifests in a unique manner. Unlike WT cells, the HSFA2 KO cells activated the UPR as part of a strategy to adapt to spaceflight, implicating that the ability to fold proteins adequately was compromised in the HSFA2 KO cells. As previously observed at the molecular level in response to a multitude of other environmental stimuli, HSFA2 was likely working to secure cellular protein homeostasis, and the inefficiency in protein folding observed in space flown HSFA2 KO cells likely resulted from the limited HSFA2-governed regulation of molecular chaperone genes. For instance, the PDI gene, ATPDIL2-3 (At2g32920), the transcription factors WRKY33 (At2g38470), and AtGATA-1 (At3g24050) along with four other genes (Ypt/Rab-GAP domain of gyp1p superfamily protein (At3g49350), SAG21 (At4g02380), transmembrane proteins 14c (At1g50740), and unknown (At1g19020)), all of which were reported to be a part of the UPR, were uniquely abundant in the HSFA2 KO cells in spaceflight [40]. Moreover, the pivotal gene of the ERAD, HRD3A (At1g18260), also showed enhanced expression in HSFA2 KO cells in spaceflight conditions, demonstrating activation of the destruction pathway for aberrant proteins. These results cumulatively suggest that the HSFA2 KO cells in spaceflight suffered from excessive aggregation of misfolded proteins and that to get rid of these misfolded and cumbersome aggregates, a degradation pathway was upregulated in order to actively remove them. 

**Figure 6:**
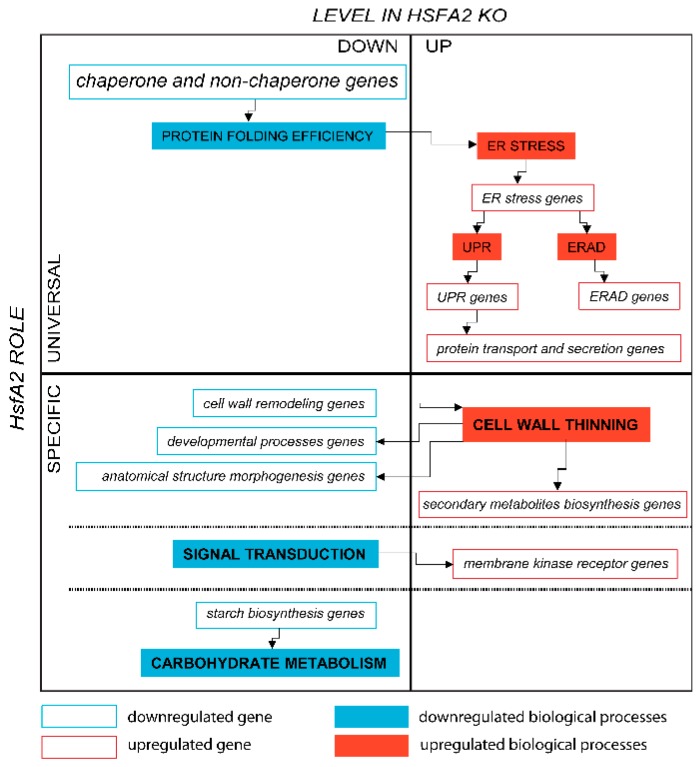
Graphical presentation of the identified processes associated with the universal stress response and specific roles of HSFA2 in spaceflight. The filled shapes represent the biological process; the unfilled shapes represent the genes; red color indicates upregulation/enhancement; blue color represents downregulation/decrease. Text in bold highlights the key specific functions.

Additionally, the genes of intracellular protein transport and secretion, such as Transducin/WD40 repeat-like superfamily protein (AT1G18830) alias SEC31-like protein transporter, target SNARE coiled-coil domain protein (AT1G29060) and the Coatomer beta subunit (AT3G15980), which functions specifically in ER to Golgi vesicle-mediated transport, were also upregulated in HSFA2 KO cells relative to WT cells in the spaceflight-adapted state [54]. The ATARFA1D (AT1G70490) gene coding Ras-related small GTP-binding family protein, known to be essential for vesicle coating and un-coating [55], was also upregulated in space-flown HSFA2 KO cells. This suggests that the protein sorting and delivery processes were impaired in space flown HSFA2 KO cells and that the over-induction of multiple vesicle-mediated protein transport genes was an attempt to circumvent an inefficient system of intracellular protein trafficking. As such, HSFA2 plays a role in processes involved when a cell physiologically adapts to spaceflight similar to its role as a universal coordinator of the stress response when plants adapt to a broad range of other environmental changes (see Figure 6). However, the expression patterns in the spaceflight-adapted state of the genes related to cell wall construction, signal transduction and starch biosynthesis in HSFA2 KO cells in comparison to WT cells imply more specific roles for HSFA2 beyond the universal stress response coordination. 

The HSFA2 KO cells in spaceflight likely have a fundamentally different architecture from the wild type cell wall, along with diminished processes of cell growth, expansion, and elongation as identified by the altered gene expression profiles. Many genes encoding proteins important to these processes are downregulated in the HSFA2 KO cells in spaceflight. For instance, the gene coding glycosyltransferase QUA1 (At3g25140), involved in homogalacturonan biosynthesis and cell adhesion, the trichome birefringence-like 45 (TBL45 (At2g30010)), which functions in the synthesis and deposition of secondary wall cellulose, and XTH4 (AT2G06850), which executes the cleavage and reconnection of cell wall xyloglucan cross-links. It was shown for many plant systems that spaceflight caused changes to the cell wall organization; thus, the finding of this study suggests that HSFA2 may have a role in the way a plant cell remodels its wall to adapt to spaceflight microgravity [14,16,20]. Given that cells deficient in *HsfA2* expression show signs of ER stress and impaired protein sorting and intracellular protein trafficking, it is possible that the proteins of the cell wall biosynthesis do not properly mature within ER, and thus fail to be properly sorted or delivered to their destination at the cell wall periphery [56,57]. However, the heat shock transcription factor of *Saccharomyces cerevisiae*, Hsf1, was shown to regulate cell wall remodeling in response to heat shock in a more specific fashion [58]. The yeast strain mutated in *Hsf1* exhibited cell wall integrity defects in the absence of stress and Hsf1 was demonstrated to regulate the expression of a set of proteins that are involved in cell wall formation and remodeling besides the universal regulation of the HSPs’ expression. The overexpression of a heat shock protein Hsp150 in *S. cerevisiae* caused upregulation of many cell wall proteins, leading to increased cell wall integrity and potentially enhanced the yeasts’ virulence [59]. Also, the *Hsf* genes were found among regulatory pathways in secondary cell wall thickening in *Medicago truncatula* using microarrays [60]. These examples further support HSFA2’s specific role in the remodeling of *Arabidopsis* cell wall architecture in the spaceflight environment. 

Another category of genes that suggested cell wall related roles for HSFA2 in spaceflight adaptation were the secondary metabolite biosynthesis genes associated with wounding (see Section 2.3.2, Figure S3 in the Supplementary Material). For instance, in spaceflight, HSFA2 KO cells overexpressed genes associated with glucosinolate biosynthesis (NSP5 nitrile specifier protein 5, At5g48180) and AOP1 (2-oxoglutarate-dependent dioxygenase, At4g03070), in anthocyanin and flavinol biosynthesis (SRG1 senescence-related gene 1, At1g17020). These genes, and others in this category of herbivore response genes, were all overexpressed in spaceflight in HSFA2 KO cells relative to WT spaceflight cells (horizontal comparison) [61,62,63]. The upregulation of these categories of genes suggests that the cells disabled in *HsfA2* responded as they would to an herbivore attack, possibly as a consequence of diminished expression of cell wall remodeling genes, resulting in a thinning of the cell wall in spaceflight-adapted cells. 

The analysis of genes with differentially expressed spaceflight vs. ground control (vertical dimension) of the WT and HSFA2 KO cells further supports the concept that the cell wall remodeling is part of the physiological adaptation to spaceflight, and that HSFA2 has a central role in this process. Both cell genotypes engaged the cell wall remodeling genes in their physiological adaptation to spaceflight but with fundamentally different outcomes: the WT cells increased the expression of these genes, while the HSFA2 KO cells reduced its expression of cell wall-related genes in spaceflight relative to their terrestrially grown counterparts (see Section 2.2.1 and Section 2.2.2, Table A1, Figure 6). The WT cells enhanced the expression of a curculin-like (mannose-binding) lectin family protein (AT1G78860) that was previously identified among the secretory cell wall *N*-glycosylated proteins in a wall-bound extracellular matrix fraction during citrus pathogen invasion [64] and is described to be associated with cell wall structural changes and signaling upon biological attack. Also overexpressed in WT cells was an unknown protein (At4g30500) linked to the cellulose biosynthetic process and subtilase (AT1G30600), which exhibits serine-type endopeptidase activity that processes the precursor proteins of the cell wall pectin modification enzymes [65]. Contrary to WT cells, the HSFA2 KO cells mostly downregulated the expression of multiple cell wall remodeling genes previously reported to be enhanced in spaceflight [14]. In spaceflight, cells in which *HsfA2* was knocked out had diminished expression of cell wall loosening and cell wall elongation genes such as GASA1 (AT1G75750), endoxyloglucan transferase XTH4 (AT2G06850), EXPL1 (AT3G45970), and EXO (AT4G08950), relative to the ground control. These cells also decreased the expression level of genes associated with cell wall composition such as PMR6 (AT3G54920), which was shown to alter the composition of plant cell wall in resistance to powdery mildew pathogen, and Glycosyl hydrolase (AT5G13980), a gene shown to degrade cell wall polysaccharides [66,67]. 

Cell wall remodeling genes associated with developmental processes and genes related to the morphogenesis of anatomical structures also showed diminished expression in the HSFA2 KO cells in spaceflight relative to its ground counterparts (see Section 2.2.2, Figure S4 drawing on the Left in the Supplementary Material). For instance, genes involved in cell elongation due to cell wall expansion such as GASA1 (At1g75750), EXLA1 (expansin-like A1, At3g45970), and GRH1 (At4g03190), a GRAS family transcription factor SCR (SCARECROW, At3g54220), which functions in asymmetric cell division, and auxin efflux carrier PIN1 (At1g73590), which is involved in the regulation of cell size, were all downregulated in the HSFA2 KO cells that had physiologically adapted to spaceflight (see Table A1, Figure S3 in the Supplementary Material) [68,69]. Additionally, 3 genes related to pollen development: 2-oxoglutarate (2OG) and Fe(II)-dependent oxygenase superfamily protein (At3g63290), ZWI ZWICHEL (At5g65930), and ATACA9, which auto inhibits Ca(2+)-ATPase 9 (At3g21180) [70,71,72], all had diminished expression in the HSFA2 KO cells adapted to the spaceflight environment. 

An essential conclusion drawn from examining the differentially expressed genes in the vertical dimension was that these genes were not differentially expressed between the two genotypes in the horizontal comparison of the ground adapted states. Therefore, the way both cell genotypes modulated the expression of cell wall genes during the physiological adaptation (vertical comparison) inevitably led to substantial differences in the distinct gene expression profiles of the WT and HSFA2 KO spaceflight-adapted cells (horizontal comparison) (Section 2.3.2). Summarizing, the HSFA2 KO cells in spaceflight exhibited the diminished cell wall remodeling processes leading presumably to cell wall thinning compared to the ground HSFA2 KO cells, therefore supporting the potential specific HSFA2 roles in the cell wall reorganization in achieving the spaceflight-adapted state.

Another specific role for HSFA2 in spaceflight adaptation is associated with the plasma membrane. Several genes associated with processes on the plasma membrane were differentially expressed in HSFA2 KO relative to WT cells in spaceflight (horizontal comparison) (see Section 2.3.2, Table A2, Figure S3 in the Supplementary Material). Examples include the overexpression of membrane kinase receptors such as: Leucine-rich repeat domain protein kinase family proteins, LRKs (At5g65240, At5g25930), STRUBBELIG-receptor family 5 (At1g78980), and BIR1 BAK1-interacting receptor-like kinase (At5g48380) [73]. Membrane receptors such as these are essential for myriad processes, including sensing chemical and physical stimuli from the environment. The abundance of the receptor proteins’ presence at the membrane translates to cell’s sensitivity to the extracellular compounds binding to these receptors and it is possible that HSFA2 KO cells modulated their responsiveness by increasing the receptors expression. It is also possible that in the absence of the HSFA2, the signal transduction from the membrane receptors is altered. The directional link between the expression of the LRR-RLK genes, enhanced expression of *HsfA2*, and many *Hsf* and *Hsp* genes and response to the exogenously administered arsenic was found in *Arabidopsis* roots, suggesting that HSFA2 may have a function in the response to toxic metals in the growth medium [74]. 

The HSFA2 KO cells in spaceflight seemed to also differ from the spaceflight WT cells (horizontal comparison) in starch biosynthesis (see Section 2.3.2). Genes central to starch biosynthesis and carbohydrate metabolism, such as phosphoglycerate/bisphosphoglyce PGM (At1g78050), isoamylase 1(At2g39930), and NDHN oxidoreductase (AT5G58260), were substantially underexpressed in the HSFA2 KO cells in spaceflight relative to WT. The reports on starch content in plants in microgravity vary with the experiment and plant species [75,76,77], but it is possible that HSFA2 plays a role in the carbohydrate metabolism and energy reserve metabolic processes in microgravity that contributes to the diverse response of plants to spaceflight.

### 3.2. HSFA2 is Much More Than a Heat Shock Factor—Things Learned from Space

Although HSFA2 is part of the Heat Shock Factor network, the molecular phenotype of the HSFA2 KO in response to spaceflight shows that the gene plays an essential role in physiological adaptation that is well beyond simple thermal metabolism. Aspects of that role are revealed by examination of the gene expression differences on the ground between HSFA2 KO and WT.

Disabling the expression of *HsfA2* caused a change in the expression of 349 genes in the ground environment. Therefore, cells that did not express *HsfA2* lacked not only one gene, but 349, as they were launched into orbit, and they all potentially contributed to the cells’ metabolic state both in the control and in the spaceflight microgravity environment. This 349-gene difference highlights the importance of recognizing the cells’ ground adapted state while looking for the differences in the tested environment which, in this study, is the environment of spaceflight. Nearly two-thirds of the genes differentially expressed between the HSFA2 KO and WT ground cells showed diminished expression in knockout cells, which could be explained by the transcription factor function of the HSFA2 (see Figure S2 in the Supplementary Material). 

The most prominent class of genes underexpressed in HSFA2 KO cells on the ground as compared to the WT is associated with protein folding (Table A2; Figure S4 drawing on the Right in the Supplementary Material). These genes included the chaperone, co-chaperone, and no-chaperone coding genes that were reported among the critical protein folding genes in *Arabidopsis* [40]. Such diminished expression of some chaperone genes was in accordance with the microarray dataset obtained from the *A. thaliana* HSFA2 KO plants (Array Express accession number E-GEOD-4760) [78]. Additionally, plants overexpressing *HsfA2* had elevated expression level of many chaperones in non-stressed conditions [79]. For instance, transgenic *Arabidopsis* plants overexpressing *HsfA2* gene overexpressed many *Hsp* genes, including 18 genes associated with cell rescue and virulence related genes in normal conditions [45]. The overexpression of *HsfA2* increased the plant’s tolerance to combined environmental stresses, while plants with knocked out *HsfA2* gene had reduced basal and acquired thermotolerance and oxidative stress tolerance [46]. These observations suggest that HSFA2 regulates the housekeeping expression of the protein folding genes not solely in response to stress.

The severe underexpression of protein folding genes in the HSFA2 KO cells on the ground that compromised the efficiency of the folding machinery was further manifested by the overexpression of the ER stress genes, notably 3 critical genes of the UPR involved in oxidative protein folding PDIs/ERO1 (alias ATPDIL1-2 (At1g77510), ATPDIL2-1 UNE5 (At2g47470) and AERO1 (At1g72280)). It seems that the *HsfA2* mutation compromised the basic maintenance of the protein folding processes that altered the ER homeostasis and that led to ER stress. As the ER stress is a hallmark of the environmental stress response, it can further explain why the HSFA2 KO cells on the ground showed enhanced expression of abiotic stress response genes, particularly genes involved in response to drought, extreme temperature, and high salinity. The genes dehydrin (AT1G54410), a hydrophobic protein RCI2A (AT3G0588), and DREB2B (AT3G11020) were all upregulated in HSFA2 KO cells in the control, ground environment [80,81,82]. In analyzing the differentially expressed ER stress genes, it seems that if a cell experiences ER stress due to an inefficiency of the protein folding machinery resulting from its genetic background, and not the environment, the cells still send a stress-response signal cascade as they would in response to damaging exogenous or environmental stimuli and activates the stress response. Therefore, the cells actively engaging in the stress response were sent to the ISS, which could explain the hypersensitive features of the HSFA2 KO cells in the spaceflight-adapted state.

HSFA2 KO underexpressed genes related to oxidative stress tolerance in the ground environment (see Table A2, Figure S4 drawing on the Right in the Supplementary Material). A strong link between HSFA2 and the oxidative stress response and tolerance is well established [83,84,85]. For instance, the gene At5g61640 coding PMSR1, a ubiquitous enzyme that repairs oxidatively damaged proteins, was underexpressed in the HSFA2 KO cells on the ground, suggesting compromised tolerance to stress at minimum oxidative stress. 

Fundamental metabolic processes can also be impacted by the environment; carbohydrate metabolism and cell wall remodeling are good examples of where HSFA2 plays a role these types of responses. Genes that are involved in the response to sucrose were also expressed at a lower level in HSFA2 KO cells in the ground-adapted state compared to WT. For instance, the Cold acclimation protein WCOR413 family (AT4G37220) gene, a canonical, highly sensitive sugar-repressed gene [86] was downregulated in HSFA2 KO cells on the ground. In accordance, DIN3 DARK INDUCIBLE 3 (AT3G06850) and MCCB 3-methylcrotonyl-CoA carboxylase (AT4G34030), both mitochondrial proteins involved in valine, leucine, and isoleucine degradation during sugar starvation [87,88] were also down-regulated. Glucose level may influence protein folding, as high glucose stimulates de novo protein synthesis, making it possible that diminished gene expression of sugar-responsive genes is part of the UPR’s strategy to minimize the level of de novo protein translation and to prevent further overload of the ER folding [89]. This connection further reinforces the link between diminished carbohydrate metabolic processes and adaptation to the spaceflight environment as observed in the HSFA2 KO cells in spaceflight. 

Cell wall remodeling is influenced by a variety of developmental and environmental cues, and it is well established that there are multiple strategies to achieve an operational goal. The extent to which HSFA2 contributes to the regulation of these strategies was revealed by comparing the expression of cell wall related genes in the HSFA2 KO to WT. It has been demonstrated in many studies that plants in space utilize genes involved in the response to pathogen attack, despite the absence of any pathogens [20]. This misconceived pathogen response could be a result of reduced gravity loading causing a distortion on the cell wall, threatening cell wall integrity similar to the physical damage that the cell wall sustains under pathogen wounding or herbivore attack. Both the WT cells and the HSFA2 KO cells differentially express cell wall-associated genes typical of pathogen attach, but not the same genes. For instance, while WT cells engaged PEN2 (AT2G44490), which triggers callose deposition in the cell wall and glucosinolate activation [90], along with two other genes (At3g43250, AT5G38980) reported to function in wounding and in defense response, the HSFA2 KO cells engaged the Disease resistance protein CC-NBS-LRR class gene (AT1G59124) [91] and NTA Seven transmembrane MLO family protein gene (AT2G17430) [92] instead. All of these genes are involved in mounting a defense in response to pathogen attack, but the gene out of this general class that is utilized depends very much on whether the genome contains an active *HsfA2* gene.

Thus, the differences in the cells’ physiological state on the ground dictate the requirement for molecular changes in order to adapt to the spaceflight environment. There was only one gene, At2g03760, coding brassinosteroid sulfotransferase 12 (SOT12), engaged in the same way in both genotypes during the physiological adaptation to spaceflight. The enhanced expression of SOT12 was correlated with actively growing cell cultures and in response to salt, osmotic stress, hormone treatments, but also in the defense response to a bacterial pathogen attack [93]. However, the physiological adaptation of the WT cells and HSFA2 KO cells shared 2 other genes, the U12-type spliceosome (At3g10400), and the Cystatin/monellin protein of unknown function (At4g03570); yet they showed an opposite change in their relative expression (see Figure 5A, Section 2.4.1). The U12 introns, although rare, are indispensable for normal growth and development of plants [94]. The U12 intron splicing is part of the non-sense-mediated mRNA decay (NMD), which is important for destroying transcripts with premature stop codons [95]. Interestingly, the *HsfA2* transcript in its alternative form *HsfA2*-II is degraded via the NMD pathway [96]. Both genes showed diminished expression in the HSFA2 KO spaceflight cells relative to their ground counterparts, while in the WT cells, the expression was enhanced in the respective comparison. However, both genes were overexpressed in the ground in HSFA2 KO cells compared to WT ground cells. As a result of the expression adjustments that occurred in the spaceflight cells, both genotypes achieved the matching expression level in the spaceflight-adapted state. This solitary example of the two genes underlines the importance of the ground-adapted state gene expression status that mandates requirements for the gene expression changes during the physiological adaptation to the novel spaceflight environment.

### 3.3. Role of HSFA2 in Spaceflight Adaptive Processes

Comparing the gene expression profiles among space-adapted genotypes allowed the identification of the genes *Required* to be expressed at certain levels in order for a plant cell to adapt to spaceflight. There were genes uniquely involved in the physiological adaptation of WT cells but not in HSFA2 KO cells, as they were already expressed at a spaceflight-adapted level in HSFA2 KO on the ground (see Figure 5C, Section 2.4.1). Thus, the WT cells needed to alter the expression level of these genes from their terrestrially observed level to express them at a level required for spaceflight survival, while the HSFA2 KO cells arrived at the ISS with these genes already expressed at the required level. For instance, the PDIL1-2 (AT1G77510) gene involved in the UPR and CUL2 (At1g02980), a ubiquitin E3 ligase of the Skp1-Cullin-F-box (SCF) class, was expressed in HSFA2 KO cells on the ground at the level required for the spaceflight-adapted state (see Table A3) [97]. The cullin protein is a part of a complex that mediates the ubiquitination and subsequent proteolytic turnover of proteins in a highly specific manner [98]. As mentioned above, with a change of the environment, de novo protein synthesis likely intensifies, increasing the chance for aberrant accumulation of unfolded proteins; thus, some elements of protein metabolism in the ER are indispensable to ensure proper ER homeostasis when the WT cells are exposed to a different environment. As already discussed, the HSFA2 KO cells had the UPR activated in a ground-adapted state due to inefficiency in the protein folding and, therefore, were already equipped with the correcting mechanisms when the environmental change occurred. 

Some other required genes were related to cell wall remodeling. For instance, a subtilase-like protein (At1g30600), with cell-wall glycoside hydrolase activity, that participates in the processing and/or turnover of cell wall proteins, was upregulated in the WT cells in the physiological adaptation to spaceflight, but this upregulation was not observed in HSFA2 KO cells, and the WT expression level in the spaceflight adaptive state matched that of the HSFA2 KO cells [64]. Cell wall protein degradation is likely to accompany any changes to the cell wall proteome related to cell wall remodeling; therefore, the expression of genes involved in proteolysis had to be at a specific level in spaceflight irrespective of the cell’s genetic background. Another required gene for spaceflight adaptation was the penetration 2 (PEN2) alias beta-glucosidase 26 (At2g44490) gene, which encodes glycosyl hydrolase. PEN2 was already mentioned as a component of the WT physiological adaptation and recruited for broad-spectrum antifungal defense responses by callose deposition in the cell wall [99]. The defense response was a biological process shared by WT and HSFA2 KO cells by the genes of the physiological adaptation to spaceflight. Identifying the defense response genes among the set of required genes supports the importance of this process in order for a cell to adapt to spaceflight.

Using the HSFA2 KO cells allowed identification of genes with expression Corrected to the wild type levels in order for cells to adapt to spaceflight. These genes were uniquely involved in HSFA2 KO physiological adaptation to spaceflight and did not have to be engaged in WT cells, as their expression was affected by the absence of a functional *HsfA2* gene in the ground-adapted state. Thus, the WT cells did not have to make any adjustments to their expression in spaceflight; only HSFA2 KO cells had to bring the expression to a certain level and thus *correct* the expression level of these genes to achieve the spaceflight-adapted state (see Figure 5D, Section 2.4.1). These corrected genes in the knockout cells define a new part of the overall landscape of essential genes to achieve a spaceflight-adapted state that would have been overlooked if only WT cells were used. Genes of the defense response, biotic and mechanical stimuli, and cell wall remodeling were already identified in plant wild type cells in spaceflight (see Section 3.2) [14,100,101], yet the utilization of HSFA2 KO cells provided additional insight into the repertoire of genes significant to these responses. The MLO7 Seven transmembrane MLO family protein (At2g17430), FBS1 F-box stress induced 1 (At1g61340), ILL6 IAA-leucine resistant (ILR)-like gene 6 (At1g44350), and AtPP2-A13 PP2-A13 phloem protein 2-A13 (At3g61060) are a few examples of individual genes which respond to wounding, mechanical stress, or external stimuli that had their expression level increased in HSFA2 KO physiological adaptation to match the WT spaceflight levels (see Table A3) [102,103,104]. Also, strictly cell wall modification genes, including PME1 pectin methylesterase 1 (At1g53840), NAD(P)-binding Rossmann-fold superfamily protein (At2g02400) that functions in lignin biosynthetic process ENODL17, and early nodulin-like protein 17 (At5g15350) associated with cell wall pectin metabolic process had their expression levels elevated in spaceflight from the level on the ground, so that it matched the WT levels in spaceflight (see Table A3).

Next, genes that encode transport proteins had their expression adjusted in HSFA2 KO cells to that of WT level, so they became evenly expressed in the spaceflight-adapted state. For instance, the transmembrane transporter genes ATATH9 ABC2 homolog (At2g40090), porin (At1g50400) family protein, and GAMMA-TIP aquaporin (At2g36830) were no longer differentially expressed in the spaceflight-adapted state between HSFA2 KO and WT cells, as their expression was adjusted in the physiological adaptation to spaceflight in HSFA2 KO cells (see Table A3). Similarly, nitrate transporter genes ENTH/ANTH/VHS (At4g40080) superfamily protein, and NRT2.7 (At5g14570) were also differentially expressed in spaceflight to match the WT expression levels of these genes. It is, however, uncertain how the loss of HSFA2 function was able to affect the transport of various moieties across the membrane in the ground environment, yet it seems that these transport processes had attained a particular level of efficiency for adaptation to the spaceflight environment. 

The spaceflight environment elicited an accumulation of several branched-chain amino acid (BCAA) degradation enzymes, the expression levels of which were also corrected from a low level of expression to a higher level of expression in the HSFA2 KO cells when compared to the expression level in WT cells in the spaceflight environment. The dark inducible 3, DIN3 gene (At3g06850) coding the subunits of the branched-chain α-ketoacid dehydrogenase complex (BCKDC), and two other genes important to small amino acid degradation, MCCB (At4g34030) coding for subunit β of methylcrotonyl-CoA carboxylase participating in BCKDC reactions, and ALDH6B2 (At2g14170) methylmalonate semi-aldehyde dehydrogenase (see Table A3). A large increase in BCAA accumulation can be observed due to environmental stress, such as darkness and carbohydrate starvation [88,105]. It is plausible that in the ground environment, the BCAA catabolism was reduced in the HSFA2 KO cells due to the overall problems with the cellular protein homeostasis, while the spaceflight environment elicited an accumulation of BCAAs, similar to that under abiotic stress, thereby raising the demand for the BCAA degradation enzyme function. Overall, using a knockout line greatly increases the list of genes that are necessary to adapt to and survive in the spaceflight environment and thereby amplifies the information gained from rare spaceflight experimentation opportunities to learn what sort of biological processes are necessary, what genes are involved in those processes, and what factors control the expression of those genes.

### 3.4. Genotypic Adaptation to Spaceflight

There were a few genes involved in the physiological adaptation to spaceflight engaged only in WT cells and not HSFA2 KO cells that resulted in the differential expression in spaceflight-adapted state between the two genotypes (see Figure 5E, Section 2.4.1). It is possible that without HSFA2 function, cells failed to properly regulate these genes’ expression to adapt to spaceflight, and therefore, the expression of these particular genes in the spaceflight adaptation is *dependent* on a functional *HsfA2* gene. For instance, the WT cells increased the expression of ATCSLC12 Cellulose-synthase (At4g07960), suggesting a higher rate of cellulose synthesis and thus cell wall reorganization processes in spaceflight-adapted state (see Table A3) [106]. Cell wall remodeling processes seemed to differ between the two genotypes, and it is possible that the increase in the cellulose synthesis in spaceflight-adapted cells somehow depends on the function of HSFA2. 

An alternative to the idea that genes engaged only in WT cells in spaceflight are dependent on a functional *HsfA2* gene is that some of the genes engaged only in WT cells and not HSFA2 KO cells were only required in WT cells but not in KO cells. In other words, it was not the inability to engage these genes that excluded them from those expressed in spaceflight, but rather simply that there was no need for them. For instance, a gene coding the membrane receptor SRF5 STRUBBELIG-receptor family 5 (At1g78980) protein was downregulated in the WT physiological adaptation to spaceflight, but not in those of the HSFA2 KO cells which resulted in lower expression level in WT spaceflight-adapted state than in HSFA2 KO cells. The STRUBBELIG (SUB) receptor mediates the signaling pathway of cell morphogenesis, the orientation of plane of cellular division, and cell proliferation [107]. It is unclear why the WT cells would diminish the expression of this membrane receptor in spaceflight, yet either a function of HSFA2 is required, or an aspect of HSFA2 KO physiology makes expression of SRF5 superfluous in the spaceflight environment. 

There were a few genes engaged only in HSFA2 KO cells and not WT cells that resulted in the differential expression in spaceflight-adapted state between the two genotypes (see Figure 5F, Section 2.4.1). It is possible that only cells with a disabled *HsfA2* gene were needed to make changes to these genes’ expression. This reflects the HSFA2 KO genotypic adaptation to spaceflight environment and likely represents the genes *Compensated* for the loss of HSFA2 function in that environment. 

Among the compensating genes with enhanced expression was the aforementioned gene of the UPR: PDI-like 2-3 (At2g32920), supporting the importance of the UPR and likely the ERAD, as well as HSFA2 KO, cells in achieving the spaceflight-adapted state (see Table A3). WT cells did not need to adjust expression of these particular genes. The ATGSTU25 glutathione S-transferase TAU 25 (At1g171800) gene was another gene with enhanced expression in HSFA2 KO cells in spaceflight. While genes encoding glutathione S-transferase TAU (e.g., ATGSTU1, ATGSTU3, ATGSTU24) were generally overexpressed in HSFA2 KO cells relative to WT cells in either environment, the HSFA2 KO cells further elevated the expression of the glutathione S-transferase genes in spaceflight to compensate for the loss of HSFA2 function in this environment. Similarly, vesicle-mediated transporter genes were among those consistently differentially expressed in HSFA2 KO relative to WT regardless of the environment, but HSFA2 KO induced additional similar genes during physiological adaptation to spaceflight and showed overexpression of such genes in the spaceflight-adapted state. Examples in this category included the Coatomer, beta’ subunit (At3g15980), Target SNARE coiled-coil domain protein (At1g29060) and Integral membrane Yip1 family protein (At2g36300) genes related to ER to Golgi and intra-Golgi vesicular traffic, suggesting that these processes needed to be compensated for the lack of HSFA2’s function in the spaceflight environment. 

The HSFA2 KO cells decreased the expression of the two calcium pump Ca(2+)-ATPase genes, ACA9 (At3g21180), and ACA2 (At4g37640) resulting in their underexpression in the spaceflight-adapted state relative to WT cells (see Table A3). Aside from their involvement in calcium homeostasis and calcium second messenger functions, these calcium pumps were also shown to participate in protein processing in the secretory pathway [108]. Interestingly, there was another gene with the same expression profile coding, the RMR1 transmembrane receptor (At5g66160) of the intra-Golgi vesicle-mediated transport. The RMR1 functions as a sorting cargo receptor for protein trafficking [109]. Thus, the HSFA2 KO cell’s strategy for adapting to the spaceflight environment included reducing the protein sorting and trafficking to the destination, possibly to compensate for the basic inefficiency in these processes when the HSFA2 was disabled. 

## 4. Materials and Methods

### 4.1. The CEL Experiment of BRIC17

For details on the CEL experiment of BRIC17, please see [28], and Figure 1, which provides images of the BRIC Hardware and cells used in these analyses. Briefly, the CEL BRIC17 experiment was launched in the Dragon capsule of SpaceX-3 Commercial Resupply Service (CRS) mission to the International Space Station (ISS) on the 1st of March 2013. The cultured cell lines (both the ground control and the spaceflight samples) were grown within 60 mm Petri plates in Petri Dish Fixation Units (PDFUs) that were housed within the Biological Research in Canisters (BRIC) hardware. The PDFU/BRIC system is a passive, air-tight, non-ventilated, non-illuminated culture system. The BRIC system is certainly not an optimal habitat for plant growth, and its limitations have been reviewed [16,110]. However, the BRIC habitat is less inimical to undifferentiated cell cultures than for photosynthetic seedlings. In addition, both spaceflight and ground control cells were each exposed to the limitations of the BRIC hardware. The spaceflight samples were launched to the ISS, while the ground controls were “launched” on a 48-h delay, and then maintained in the ISS Environmental Simulator (ISSES) chamber at Kennedy Space Center (KSC). The 48-h delay facilitated the downlink of ISS environmental data for programming the ISSES chamber. The downlinked data included environmental conditions on the ISS that were duplicated in the ISSES. Cells were fixed within the BRIC canisters on the ISS with RNAlater^TM^ (Ambion) on the 10th day in orbit, and the ground controls were fixed 48 h later. Twenty-four hours after fixation, the entire BRIC was moved to the Minus Eighty-degree Laboratory Freezer for ISS (MELFI) until the spaceflight samples were transported back to Earth, and the ground control samples were transferred to a standard laboratory −80 °C freezer. The total RNA was extracted from the spaceflight samples and the corresponding ground control samples and subjected to microarrays. 

### 4.2. Tissue Culture Cell Lines

*Arabidopsis* callus cultures were established de novo, and each cell line was initiated simultaneously approximately 6 months before launch. Cells derived from hypocotyls were grown and maintained on plates with solid media containing MS salts (4.33 g/L), 3% sucrose (30 g/L), MS vitamins (1 mL of 1000× solution), 2,4-Dichlorophenoxyacetic acid (2,4-D) (0.3 mL/L), 0.5% agar (5 g/L) and kinetin (0.2 mg/L) until dedifferentiated into callus. The callus cells were then transferred to the standard liquid media containing MS salts (4.33 g/L), 3% sucrose (30 g/L), MS vitamins (1 mL of 1000× solution), and 2,4-D (0.5 mL/L) and maintained in a sterile continuous cell suspension culture. Two cell lines, each of Col-0 ecotype, were the subjects of this study: wild type (WT), and a knockout line generated from the *HsfA2* T-DNA insertion (SALK_008978C) (HSFA2 KO). The SALK line was obtained through The Arabidopsis Information Resource (TAIR), (www.arabidopsis.org) [111].

### 4.3. Preparation of BRIC17 CEL Cell Culture Plates

Liquid suspension cells growing in log phase were transferred to solid media two and a half days prior to turning over the payload in preparation for launch. The liquid media was decanted, washed once with fresh liquid media, and then decanted again. A sterile scoop was used to place about 1 g of cells on the surface of a 60 mm Petri plate (Falcon, Becton Dickinson Labware, Franklin Lakes, NJ, USA) containing 6.5 mL nutrient agar media (MS salts (4.33 g/L), 3% sucrose (30 g/L), MS vitamins, 2,4-D 2-(N-morpholino)ethanesulfonic acid (MES) buffer (0.5 g/L), 0.8% agar (8 g/L)). The cells were then dispersed evenly across the surface. All plate manipulations were conducted under sterile conditions in a laminar flow hood to assure sterility of both the interior and exterior of plates. Plates were put into a sterile Nalgene™ BioTransport Carrier (Thermo Scientific, Waltham, MA, USA), each layer of plates separated with a sterile non-skid plastic insert. The BioTransporter was then sealed with gas-permeable tape (3M), wrapped in Steri-Wrap™ autoclave wraps (Fisher, Waltham, MA, USA) and then driven to KSC. The BRIC17 CEL experiment was turned over to payload engineers in the SSPF (Space Station Processing Facility) at KSC 48 h before the scheduled launch time. 

### 4.4. RNA Extractions

Total RNA was extracted using Qiashredder and RNAeasy™ kits from QIAGEN (QIAGEN Sciences, Germantown, MD, USA) according to the manufacturer’s instructions. Residual DNA was removed by performing an on-column digestion using an RNase Free DNase (QIAGEN GmbH, Hilden, Germany). Integrity of the RNA was evaluated using the Agilent 2100 BioAnalyzer (Agilent Technologies, Santa Clara, CA, USA). 

### 4.5. Microarrays

cDNA was synthesized using Ovation Pico WTA System (NuGEN Technologies Inc., Redwood City, CA, USA) and labeled using Encore Biotin Module (NuGEN Technologies Inc.,Redwood City, CA, USA). Amplified and labeled cDNA (5 µg/sample) was fragmented and hybridized with rotation onto Affymetrix GeneChip Arabidopsis ATH1 Genome Arrays for 16 h at 45 °C. Arrays were washed on a Fluidics Station 450 (Affymetrix, Santa Clara, CA, USA) with the Hybridization Wash and Stain Kit (Affymetrix) and the Washing Procedure FS450_0004. Scanning was performed using Affymetrix GeneChip Scanner 3000 7G. For both spaceflight and ground control, 5 plates of WT and 3 plates of HSFA2 KO were analyzed as biological replicates. 

#### 4.5.1. Microarray Data Analysis

Affymetrix^®^ Expression Console™ Software (Version 1.3) was used to generate. CEL files for each RNA hybridization. All analysis was performed in R 3.0.0 [112]. Background adjustment, summarization, and quantile normalization were performed using Limma package [113]. Normalization was made using the Affymetrix MAS 5.0 normalization algorithm [114]. Data quality was assessed using the arrayQualityMetrics package and various QC charts (Density and Intensity plot, NUSE, RLE, and RNA Degradation Plot). Probes that had signals absent in all samples were removed. For each replicate array, each probe-set signal value from spaceflight samples was compared to the probe-set signal value of ground control samples to give gene expression ratios. Differentially expressed genes were identified using the Limma package with a Benjamini and Hochberg false discovery rate multiple testing correction. Genes were considered as differentially expressed with stringent criteria at *P*-value < 0.01, abs Fold Change > 2 (−1< FC log_2_ > +1; labeled as log_2_ Fold Change) unless stated otherwise.

#### 4.5.2. Comparison Groups

The groups outlined in the concept of operations and comparisons were established and abbreviated with a combination of two capital letters and a short version of the cell line name in superscript (see Figure 2A). Letters: G—Ground Control, F—Spaceflight; Superscripts: Wt—WT cells, Hsf—HSFA2 KO cells.

### 4.6. Functional Gene Categorization—Gene Ontology Annotations

Gene function was annotated by associations of controlled vocabularies or keywords to data objects (Gene Ontology, GO). Multiple GO toolkits of this controlled vocabulary system were used to collect annotations of gene function. Various lists of gene names were created, and enrichment GO terms were searched after statistical test from pre-calculated backgrounds. All three aspects of gene products (molecular function, biological process, and subcellular location) described by GO controlled vocabularies were considered. A significance level of 0.05 and five genes as minimum number of mapping entries were implemented for the analysis parameters in the following tools:

- **AgriGO**—An integrated web-based GO analysis toolkit for the agricultural community AgriGO was used [115]. AgriGO query criteria were as follows: Singular Enrichment Analysis (SEA), *Arabidopsis* genemodel (TAIR9) precomputed background, Fisher was selected as the statistical test method of choice with the NOT-adjust multi-test adjustment method; significance level was set at 0.01 or 0.05, minimum number of mapping of entries was set at 5, plant GO slim was selected from other Gene ontology types. For Parametric Analysis of Gene Set Enrichment (PAGE), the selected species was *Arabidopsis thaliana*, NOT-adjust was selected for multi-test adjustment method, significance level was set at 0.1, minimum number of mapping of entries was set at 10, and Plant GO slim was selected from other Gene ontology types.
- **AmiGO**—If needed, the GO database was accessed through the AmiGO query tool.
- **ATTED-II**—The ATTED-II database of coexpressed genes, developed to identify functionally related genes in *Arabidopsis*, was also used [116]. The make gene function table function was implemented to retrieve organized information on gene function (based on TAIR annotation) and subcellular localization (as predicted by TargetP and WOLF PSORT).
- **gProfiler**—A web-based toolset for functional profiling of gene lists was used [117]. *Arabidopsis thaliana* was the selected organism with most of default options except where the Benjamini-Hochberg FDR significance threshold was selected.

## 5. Conclusions

Conclusions from these data concern non-thermal roles for the heat shock factors in the physiological adaptation of plant cells to spaceflight. First, the heat shock transcription factor HSFA2, and likely the whole HSF network, has important roles in the physiological adaptation to spaceflight through regulation of cell wall and plasma membrane signaling, as well as starch biosynthesis. Second, disabling *HsfA2* leads to activation of the endoplasmic reticulum stress response and the unfolded protein stress response and dramatically alters adaptation to spaceflight. Without HSFA2, a much larger change in gene expression is required for spaceflight adaptation. Third, the endoplasmic reticulum and unfolded protein stress responses define the HSFA2 KO cell physiological state regardless of the environment, likely because of the deficiency in the chaperone-mediated protein folding machinery. Fourth, the HSFA2 KO cells helped to unravel the *HsfA2*-dependent genes of the wild type adaptation to spaceflight by identifying a set of genes with a required expression level for a cell to achieve the spaceflight-adapted state, thus suggesting that genetic approaches provide additional insights beyond those revealed by differential gene expression in WT cells.

## Data Availability

The microarray data are publicly available from GEO under accession number: GSE95388, link: https://www.ncbi.nlm.nih.gov/geo/query/acc.cgi?acc=GSE95388. In addition, GeneLab accession number (TBD).

## Supplementary Materials

Supplementary materials can be found at http://www.mdpi.com/1422-0067/20/2/390/s1.

## Appendix A. Gene Expression Tables

**Table A1:** The selected significant GO terms assigned with AgriGO, gProfiler and ATTED-II to 78 genes of the physiological adaptation to spaceflight in WT cells, and 221 genes of the physiological adaptation to spaceflight in HSFA2 KO cells. In some instances, the gene duplicates within ontology were removed and assigned to the most specific available GO term class. Cells highlighted in red indicate upregulation; in green indicate downregulation. Cells marked with “x” indicate no statistically significant differential expression.

ONT	GO Term Name				
	Transcript ID	Gene Symbol	Gene Description	F^Wt^ : G^WT^ FC log_2_	F^Hsf^ : G^Hsf^ FC log_2_
BP	defense response to other organism GO:0098542				
	AT2G03760	ST1	sulphotransferase 12	1.1	1.0
	AT5G38980		unknown	3.3	X
	At3g43250		unknown in wounding	2.6	X
	AT4G02460	PMS1	DNA mismatch repair protein, putative	2.2	
	AT2G44490	PEN2	Glycosyl hydrolase superfamily protein	1.4	X
	AT1G59124		Disease resistance protein (CC-NBS-LRR class)	X	2.4
	AT2G17430	NTA	Seven transmembrane MLO family protein	X	1.9
	AT4G35770	SEN1	Rhodanese/Cell cycle control phosphatase	X	1.8
	AT3G61060	PP2-A13	phloem protein 2-A13	X	1.5
	AT3G09940	MDHAR	monodehydroascorbate reductase	X	1.4
CC	plant-type cell wall GO:0009505				
	AT1G30600		Subtilase family protein	2.5	X
	At4g30500		unknown in cellulose biosynthetic process	2.4	X
	AT2G44490	PEN2	Glycosyl hydrolase superfamily protein	1.4	X
	AT1G78860		curculin-like (mannose-binding) lectin	1.3	X
	AT5G44130	FLA13	FASCICLIN-like arabinogalactan protein 13 precursor	−2.1	X
	AT2G06850	XTH4	xyloglucan endotransglucosylase/hydrolase 4	X	−2.4
	AT5G51550	EXL3	EXORDIUM like 3	X	−2.1
	AT5G13980		Glycosyl hydrolase family 38 protein	X	−1.6
	AT3G45970	EXPL1	expansin-like A1	X	−1.4
	AT1G15390	PDF1A	peptide deformylase 1A	X	−1.4
	AT1G75750	GASA1	GAST1 protein homolog 1	X	−1.4
	AT4G08950	EXO	EXO Exordium, Phosphate-responsive 1 protein	X	−1.1
	AT1G73590	PIN1	Auxin efflux carrier family protein	X	−1.0
BP	regulation of biological process GO:0050789 regulation of metabolic process GO:0019222 regulation of cellular metabolic process GO:0031323				
	AT3G62080		SNF7 family protein	2.6	X
	AT5G48560		basic helix-loop-helix (bHLH) DNA-binding	2.4	X
	AT5G01920	STN8	Protein kinase superfamily protein	2.4	X
	AT2G46225	ABIL1	ABI-1-like 1	2.2	X
	AT5G56270	WRKY2	WRKY DNA-binding protein 2	1.9	X
	AT1G27370	SPL10	squamosa promoter binding protein-like 10	1.7	X
	AT5G62710		Leucine-rich repeat protein kinase family protein	1.4	X
	AT5G05130		DNA/RNA helicase protein	1.1	X
	AT5G07580		Integrase-type DNA-binding superfamily protein	−3.3	X
	AT4G21380	RK3	receptor kinase 3	−2.9	X
	AT2G35530	bZIP16	basic region/leucine zipper transcription factor 16	−2.7	X
	AT1G78980	SRF5	STRUBBELIG-receptor family 5	−2.4	X
	AT1G78080	WIND1	related to AP2 4	−1.2	X
	AT4G36530		alpha/beta-Hydrolases superfamily protein	−1.2	X
CC	endomembrane system GO:0012505 Golgi apparatus GO:0005794				
	At3g18260		Reticulon family protein	3.1	X
	At1g77510	PDIL1-2	PDI-like 1-2 protein disulfide isomerase-like 1-2	1.2	X
	At2g03760	ST1	sulphotransferase 12	1.1	X
	AT5G19070		SNARE associated Golgi protein family	−2.6	X
	At2g22900		Galactosyl transferase GMA12/MNN10	−2.4	X
	At4g36640		Sec14p-like phosphatidylinositol transferprotein	−1.8	X
	At2g32720	CB5-B	cytochrome B5 isoform B	−1.5	X
	AT1G19970		ER lumen protein retaining receptor protein	−1.4	X
	AT2G43240		Nucleotide-sugar transporter family protein	−1.3	X
BP	cellular response to sucrose starvation GO:0043617				
	AT3G06850	DIN3	2-oxoacid dehydrogenases acyltransferases	X	1.2
	AT3G13450	DIN4	branched chain alpha-keto acid dehydrogenase E1 beta	X	1.3
	AT1G21400	E1α	Thiamin diphosphate-binding fold (THDP-binding)	X	1.6
	Valine, leucine and isoleucine degradation GO:0009083				
	AT3G06850	DIN3	2-oxoacid dehydrogenases acyltransferase family protein	X	1.2
	AT3G13450	DIN4	branched chain alpha-keto acid dehydrogenase E1 beta	X	1.3
	AT1G21400	E1α	Thiamin diphosphate-binding fold (THDP-binding)	X	1.6
	AT2G14170	ALDH6B2	aldehyde dehydrogenase 6B2	X	1.2
	At4g34030	MCCB	subunit β of methylcrotonyl-CoA carboxylase (MCCB)	X	1.1
CC	plasma membrane GO:0005886				
	AT5G67130		PLC-like phosphodiesterases superfamily	X	−2.7
	AT3G21180	ATACA9	autoinhibited Ca(2+)-ATPase 9	X	−2.7
	AT5G65440			X	−1.9
	AT5G15350	ENODL17	early nodulin-like protein 17	X	−1.7
	AT5G13980		Glycosyl hydrolase family 38 protein	X	−1.6
	AT3G54920	PMR6	Pectin lyase-like superfamily protein	X	−1.5
	AT1G17620		Late embryogenesis abundant (LEA)	X	−1.2
	AT5G03700		D-mannose binding lectin protein	X	−1.1
	AT1G73590	PIN1	Auxin efflux carrier family protein	X	−1.0
BP	developmental process GO:0032502 anatomical structure morphogenesis GO:0009653				
	AT2G35340	MEE29	helicase domain-containing protein	X	−3.2
	AT3G63290		2-oxoglutarate (2OG) and Fe(II)-dependent oxygenase	X	−3.1
	AT5G65930	ZWI	kinesin-like calmodulin-binding protein (ZWICHEL)	X	−3.0
	AT4G02460	PMS1	DNA mismatch repair protein, putative	X	−2.9
	AT3G47450	RIF1	P-loop containing nucleoside triphosphate hydrolase	X	−2.8
	AT3G21180	ATACA9	autoinhibited Ca^2+^-ATPase 9	X	−2.7
	AT2G06850	XTH4	xyloglucan endotransglucosylase/hydrolase 4	X	−2.4
	AT4G15570	MAA3	P-loop containing nucleoside triphosphate hydrolase	X	−1.8
	AT3G54220	SCR	SCR Scarecrow alias SGR1 Shoot Gravitropism	X	−1.7
	AT3G45970	EXPL1	expansin-like A1	X	−1.4
	AT1G75750	GASA1	GAST1 protein homolog 1	X	−1.4
	AT4G03190	GRH1	GRR1-like protein 1	X	−1.1
	AT1G73590	PIN1	Auxin efflux carrier family protein	X	−1.0

**Table A2:** The selected significant GO terms assigned with AgriGO, gProfiler, and ATTED-II to 349 genes significantly differentially expressed in the ground-adapted state between HSFA2 KO and WT cells, and 220 genes significantly differentially expressed in the spaceflight-adapted state between HSFA2 KO and WT cells. In some instances, the gene duplicates within ontology were removed and assigned to the most specific available GO term class. Cells highlighted in red indicate upregulation; in green indicate downregulation. Cells marked with “x” indicate no statistically significant differential expression.

ONT	GO Term Name				
	Transcript ID	Gene Symbol	Gene Description	G^Hsf^ : G^Wt^ FC log_2_	F^Hsf^ : F^Wt^ FC log_2_
BP	response to stimulus GO:0050896 defense response to other organism GO:0098542				
	AT2G24570	WRKY17	WRKY DNA-binding protein 17	2.1	X
	AT2G44490	PEN2	Glycosyl hydrolase superfamily protein	1.7	X
	AT1G66760		MATE efflux family protein	1.6	X
	AT1G72900		Toll-Interleukin-Resistance (TIR) domain-containing protein	1.2	X
	AT1G64790	ILA	ILITYHIA	1.2	X
	AT2G43535		Scorpion toxin-like knottin superfamily protein	1.1	X
	AT5G07010	ST2A	sulfotransferase 2A	1.1	X
	AT1G58360	NAT2	amino acid permease 1	1.0	X
	AT1G71140		MATE efflux family protein	1.1	1.7
	AT1G19020			X	1.1
	AT1G50740		Transmembrane proteins 14C	X	1.1
	AT3G49350		Ypt/Rab-GAP domain of gyp1p superfamily protein	X	2.4
	AT3G60420		Phosphoglycerate mutase family protein	X	1.5
	AT4G02380	SAG21	senescence-associated gene 21	X	1.2
	AT5G25930		Protein kinase family protein with LRR domain	X	1.0
	AT5G48380	BIR1	BAK1-interacting receptor-like kinase 1	X	1.2
	response to water deprivation GO:0009414 (high salinity)				
	AT3G05880	RCI2A	Low temperature and salt responsive protein family	1.5	X
	AT3G11020	DREB2B	DRE/CRT-binding protein 2B	1.1	X
	AT1G54410		dehydrin family protein	1.3	X
	response to endoplasmic reticulum stress (ER stress) GO:0034976 response to unfolded protein response (UPR) GO:0006986 response to misfolded protein GO:0051788 response to topologically incorrect protein GO:0035966				
	At1g72280	AERO1	disulfide bond formation protein	2.9	X
	AT5G38900		Thioredoxin superfamily protein	2.8	X
	AT1G77510	PDIL1-2	PDI-like 1-2 disulfide isomerase	2.0	X
	AT2G47470	PDIL2-1/UNE5	thioredoxin family protein	1.1	X
	AT2G29470	GSTU3	glutathione S-transferase tau 3	1.7	1.8
	AT2G29490	GSTU1	glutathione S-transferase TAU 1	1.4	1.5
	AT1G17170	GSTU24	glutathione S-transferase TAU 24	1.2	1.4
	At2g38470	WRKY33	WRKY-type DNA binding protein	1.2	1.2
	At3g49350	---	Ypt/Rab-GAP domain of gyp1p protein	X	2.4
	At2g32920	PDIL2-3	PDI-like 2-3 disulfide isomerase	X	1.5
	At4g02380	SAG21	senescence-associated gene 21	X	1.2
	At1g50740	---	Transmembrane proteins 14C	X	1.1
	At3g24050	AtGATA-1	GATA transcription factor 1 (AtGATA-1)	X	1.1
	At1g19020	---	Expressed protein	X	1.1
	endoplasmic reticulum associated degradation (ERAD) GO:0036503				
	AT1G18260	HRD3A	HCP-like superfamily protein	X	1.2
	protein localization GO:0008104 establishment of protein localization GO:0045184 protein transport GO:0015031 vesicle-mediated transport GO:0016192				
	AT1G29310		SecY protein transport family protein	1.0	1.7
	AT3G44340	CEF	clone eighty-four	1.0	1.1
	AT1G18830		Transducin/WD40 repeat-like superfamily protein	X	1.2
	AT1G29060		Target SNARE coiled-coil domain protein	X	1.5
	AT1G50740		Transmembrane proteins 14C	X	1.1
	AT1G70490	ATARFA1D	Ras-related small GTP-binding family protein	X	1.7
	AT3G15980		Coatomer, beta’ subunit	X	1.1
	AT3G49350		Ypt/Rab-GAP domain of gyp1p superfamily protein	X	2.4
	AT4G02380	SAG21	senescence-associated gene 21	X	1.2
	AT5G25930		Protein kinase family protein with LRR domain	X	1.0
	localization GO:0051179 transport GO:0006810 establishment of localization GO:0051234				
	AT4G24120	YSL1	YELLOW STRIPE like 1	2.5	X
	AT2G44490	PEN2	Glycosyl hydrolase superfamily protein	1.7	X
	AT5G39040	TAP2	transporter assoc. with antigen processing protein 2	1.6	X
	AT1G66760		MATE efflux family protein	1.6	X
	AT1G30400	MRP1	multidrug resistance-associated protein 1	1.5	X
	AT3G53480	PIS1	pleiotropic drug resistance 9	1.5	X
	AT2G15880		Leucine-rich repeat (LRR) family protein	1.3	X
	AT3G48970		Heavy metal transport/detoxification protein	1.3	X
	AT3G12520	SULTR4;2	sulfate transporter 4;2	1.2	X
	AT1G58360	NAT2	amino acid permease 1	1.0	X
	AT2G41700	AtABCA1	ATP-binding cassette A1	1.0	X
	AT5G27150	NHX1	Na+/H+ exchanger 1	1.0	X
	AT1G71330	NAP5	non-intrinsic ABC protein 5	1.5	1.2
	AT1G78570	ROL1	rhamnose biosynthesis 1	1.4	1.1
	AT1G71140		MATE efflux family protein	1.1	1.7
	AT5G54860		Major facilitator superfamily protein	1.1	1.5
	AT2G45180		Bifunctional inhibitor/lipid-transfer protein	X	2.9
	AT4G27840		SNARE-like superfamily protein	X	2.7
	AT5G19070		SNARE associated Golgi protein family	X	2.7
	AT2G29940	PDR3	pleiotropic drug resistance 3	X	2.4
	AT5G47730		Sec14p-like phosphatidylinositol transfer protein	X	2.0
	AT2G43240		Nucleotide-sugar transporter family protein	X	1.6
	AT2G36300		Integral membrane Yip1 family protein	X	1.2
	AT1G07030		Mitochondrial substrate carrier family protein	X	1.2
	secondary metabolic process GO:0019748				
	AT2G29470	GSTU3	glutathione S-transferase tau 3	1.7	1.8
	AT1G78570	ROL1	rhamnose biosynthesis 1	1.4	1.1
	AT2G29490	GSTU1	glutathione S-transferase TAU 1	1.4	1.5
	AT1G17170	GSTU24	glutathione S-transferase TAU 24	1.2	1.4
	AT2G38470	WRKY33	WRKY DNA-binding protein 33	1.2	1.2
	AT4G03070	AOP1.1	2-oxoglutarate (2OG) and Fe(II)-dependent oxygenase	X	3.7
	AT1G17180	GSTU25	glutathione S-transferase TAU 25	X	1.9
	AT5G48180	NSP5	nitrile specifier protein 5	X	1.4
	AT1G17020	SRG1	senescence-related gene 1	X	1.1
CC	plasma membrane GO:0005886 (plasma membrane receptor kinases)				
	AT1G78980	SRF5	STRUBBELIG-receptor family 5	X	2.2
	AT5G65240		Leucine-rich repeat protein kinase family protein	X	1.9
	AT5G48380	BIR1	BAK1-interacting receptor-like kinase 1	X	1.2
	AT5G25930		Protein kinase family protein with LRR domain	X	1.0
BP	response to stimulus GO:0050896 response to stress GO:0006950				
	protein folding (chaperones)				
	AT2G26150	HSFA2	heat shock transcription factor A2	−7.3	−6.8
	At3g08910		DNAJ heat shock family protein	−3.2	X
	At4g26780	MGE2	MGE2 Co-chaperone GrpE family protein	−2.5	X
	At1g80030		DnaJ protein	−1.4	X
	At5g37670		low-molecular-weight heat shock protein	−1.2	X
	At4g28480		DNAJ heat shock family protein	−1.1	X
	protein folding (non-chaperones)				
	At1g44414		unknown	−1.5	X
	At1g03070		Bax inhibitor-1 family protein	−1.3	X
	At3g15180		ARM repeat superfamily protein	−1.3	X
	At5g48580	FKBP15-2	peptidyl-prolyl cis-trans isomerase-like protein	−1.1	X
	At4g23493		unknown	−1.1	X
	response to oxidative stress GO:0006979 response to reactive oxygen species GO:0000302 response to hydrogen peroxide GO:0042542				
	AT5G19875			−3.0	X
	AT1G76080	CDSP32	chloroplastic drought-induced stress protein 32 kD	−2.3	X
	AT4G08920	OOP2	cryptochrome 1	−2.0	X
	AT3G16500	PAP1	phytochrome-associated protein 1	−1.9	X
	AT4G34020	DJ1C	Class I glutamine amidotransferase-like protein	−1.7	X
	AT4G35770	SEN1	Rhodanese/Cell cycle control phosphatase	−1.6	X
	AT2G47180	GolS1	galactinol synthase 1	−1.5	X
	AT3G20340			−1.4	X
	AT4G37530		Peroxidase superfamily protein	−1.3	X
	AT4G27670	HSP21	heat shock protein 21	−1.3	X
	AT3G25530	GR1	glyoxylate reductase 1	−1.2	X
	AT5G61640	PMSR1	peptidemethionine sulfoxide reductase 1	−1.2	X
	AT2G14170	ALDH6B2	aldehyde dehydrogenase 6B2	−1.1	X
	AT3G53260	PAL2	phenylalanine ammonia-lyase 2	−1.1	X
	AT1G52760	LysoPL2	lysophospholipase 2	−1.0	X
	response to sucrose stimulus GO:0009744				
	AT2G17880		Chaperone DnaJ-domain superfamily protein	−3.0	X
	At3g61060	PP2-A13	phloem protein 2-A13	−2.1	X
	AT4G37220		Cold acclimation protein WCOR413 family	−1.9	X
	At3g24190		Protein kinase superfamily protein	−1.7	X
	AT3G06850	DIN3/LTA1	2-oxoacid dehydrogenases acyltransferase	−1.2	X
	cellular amino acid and derivative metabolic process GO:0006519				
	At5g65010	ASN2	asparagine synthetase 2	−2.9	X
	At2g02400		NAD(P)-binding Rossmann-fold superfamily protein	−1.6	X
	At3g07630	AtADT2	arogenate dehydratase 2	−1.4	X
	At5g24530	DMR6	2-oxoglutarate (2OG) and Fe(II)-dependent oxygenase	−1.4	X
	At1g73500	MKK9	MAP kinase kinase 9	−1.2	X
	At4g34030	MCCB	3-methylcrotonyl-CoA carboxylase	−1.1	X
	At5g48220		Aldolase-type TIM barrel family protein	−1.1	X
CC	cell wall GO:0005618 external encapsulating structure GO:0030312				
	AT1G15020	QSOX1	quiescin-sulfhydryl oxidase 1	X	−3.1
	AT1G76020		Thioredoxin superfamily protein	X	−2.8
	At2g30010	TBL45	trichome birefringence-like 45	X	−2.4
	AT3G25140	QUA1/GAUT8	galacturanosyl transferase 8	X	−2.0
	AT2G06850	XTH4	xyloglucan endotransglucosylase/hydrolase 4	X	−2.0
	At4g07960	CSLC12	CSL12 Cellulose-synthase-like C12	X	−2.0
	At5g50030		Plant invertase/pectin methylesterase inhibitor protein	X	−1.9
	At1g16530	ASL9	assymetric leaves 2 like 9	X	−1.7
	AT1G75830	PDF1.1	low-molecular-weight cysteine-rich 67	X	−1.1
	AT2G02100	PDF2.2	low-molecular-weight cysteine-rich 69	X	−1.1
	AT5G64260	EXL2	EXORDIUM like 2	X	−1.1
	plastid GO:0009536				
	AT3G19120		PIF / Ping-Pong family of plant transposases	X	−3.4
	AT5G04810		pentatricopeptide (PPR) repeat-containing protein	X	−3.0
	AT5G16810		Protein kinase superfamily protein	X	−2.6
	AT5G47870	RAD52-2B		X	−2.4
	AT1G54350	ABCD2	ABC transporter family protein	X	−2.2
	AT1G32440	PKp3	plastidial pyruvate kinase 3	X	−2.2
	AT1G14410	WHY1	ssDNA-binding transcriptional regulator	X	−1.8
	AT3G52150		RNA-binding (RRM/RBD/RNP motifs) family protein	X	−1.6
	AT3G20930		RNA-binding (RRM/RBD/RNP motifs) family protein	X	−1.5
	AT2G20690		lumazine-binding family protein	X	−1.0
	AT2G45990			X	−1.0
	energy reserve metabolic process GO:0006112 (starch biosynthetic process GO:0019252)				
	AT3G47450	RIF1	P-loop containing nucleoside triphosphate hydrolase	X	−2.6
	AT5G58260	NdhN	oxidoreductase	X	−2.4
	At1g78050	PGM	phosphoglycerate/bisphosphoglyce	X	−2.2
	AT2G39930	ISA1	isoamylase 1	X	−1.7
	AT4G39550		Galactose oxidase/kelch repeat superfamily protein	X	−1.3

**Table A3:** The selected individual genes with a corresponding functional annotation from the Required, Corrected, *HsfA2*-dependent; WT genotypic adaptation, or Compensated; HSFA2 KO genotypic adaptation presented on Figure 5C–F. Cells highlighted in red indicate upregulation; in green indicate downregulation. Cells marked with “x” indicate no statistically significant differential expression.

Category	Transcript ID	Gene Symbol	Gene Description	Functional Annotation	F^Wt^ : G^WT^ FC log_2_	F^Hsf^ : G^Hsf^ FC log_2_	G^Hsf^ : G^Wt^ FC log_2_	F^Hsf^ : F^Hsf^ FC log_2_
Required	At1g02980	CUL2	cullin 2	proteolytic turnover	2.5	X	3.0	X
	At3g18260	---	Reticulon family protein	ER	3.1	X	3.7	X
	At1g77510	ATPDIL1-2	PDI-like 1-2	ER stress, UPR	1.2	X	2.0	X
	At1g30600		Subtilase family protein	cell wall	2.5	X	2.7	X
	At2g44490	PEN2	Penetration 2, BGLU26 Beta Glucosidase 26	defense response, response to wounding	1.4	X	1.7	X
Corrected	At5g40470		RNI-like superfamily protein	response to wounding, mechanical stress	X	2.1	−2.1	X
	At1g44350	ILL6	ILL6 IAA-leucine resistant (ILR)-like gene 6	response to wounding, mechanical stress	X	1.9	−2.0	X
	At2g17430	MLO7	Seven transmembrane MLO family protein	response to wounding, mechanical stress	X	1.9	−1.7	X
	At3g61060	AtPP2-A13	PP2-A13 phloem protein 2-A13	response to wounding, mechanical stress	X	1.5	−2.1	X
	At4g38550		phospholipase-like protein (PEARLI 4) family	response to wounding, mechanical stress	X	1.4	−1.3	X
	At1g61340		FBS1 F-box stress induced 1	response to wounding, mechanical stress	X	1.0	−1.8	X
	At2g02400		NAD(P)-binding Rossmann-fold superfamily protein	cell wall	X	2.0	−1.6	X
	At1g53840	ATPME1	PME1 pectin methylesterase 1	cell wall	X	1.1	−1.4	X
	At5g15350	ENODL17	early nodulin-like protein 17	cell wall	X	−1.7	1.0	X
	At2g36830	GAMMA-TIP	GAMMA-TIP gamma tonoplast intrinsic protein	transmembrane transporter genes	X	3.1	−3.5	X
	At1g50400		porin family protein	transmembrane transporter genes	X	2.2	−2.0	X
	At2g40090	ATATH9	ABC2 homolog 9	transmembrane transporter genes	X	1.1	−1.0	X
	At5g14570	ATNRT2.7	NRT2.7 high affinity nitrate transporter	nitrate transporters genes	X	2.4	−2.6	X
	At4g40080		ENTH/ANTH/VHS superfamily protein	nitrate transporters genes	X	2.1	−2.3	X
	At3g06850	DIN3	DIN3 dark inducible 3, BCE2	valine, leucine and isoleucine degradation	X	1.2	−1.2	X
	At2g14170	ALDH6B2	ALDH6B2 aldehyde dehydrogenase 6B2	valine, leucine and isoleucine degradation	X	1.2	−1.1	X
	At4g34030	MCCB	MCCB 3-methylcrotonyl-CoA carboxylase	valine, leucine and isoleucine degradation	X	1.1	−1.1	X
HsfA2-Dependent WT genotypic adaptation	At4g07960	ATCSLC12	Cellulose-synthase-like C12	cell wall	1.9	X	X	−2.0
	At1g78980	SRF5	SRF5 STRUBBELIG-receptor family 5	plasma membrane receptor kinase	−2.4	X	X	2.2
	At5g19070	---	SNARE associated Golgi protein family	vesicle-mediated transport	−2.6	X	X	2.7
	At2g43240	---	Nucleotide-sugar transporter family protein	transport	−1.3	X	X	1.6
Compensated HSFA2 KO genotypic adaptation	At2g32920	ATPDIL2-3	PDI-like 2-3	ER stress, UPR	X	1.0	X	1.5
	At1g17180	ATGSTU25	glutathione S-transferase TAU 25	UPR	X	1.7	X	1.9
	At3g15980	---	Coatomer, beta’ subunit	vesicle-mediated transport	X	1.7	X	1.7
	At1g29060	---	Target SNARE coiled-coil domain protein	vesicle-mediated transport	X	1.3	X	1.5
	At2g36300	---	Integral membrane Yip1 family protein	vesicle-mediated transport	X	1.0	X	1.2
	At3g12180	---	Cornichon family protein	intra-Golgi vesicle-mediated transport	X	−2.7	X	−2.0
	At5g66160	RMR1	receptor homology, transmembrane domain ring H2	intra-Golgi vesicle-mediated transport	X	−1.6	X	−1.7
	At3g21180	ACA9	autoinhibited Ca(2+)-ATPase 9	calcium pump	X	−2.7	X	−2.6
	At4g37640	ACA2	calcium ATPase 2	calcium pump	X	−2.2	X	−1.7
