# Supplementary material for: HSFA2 Functions in the Physiological Adaptation of Undifferentiated Plant Cells to Spaceflight
Source: Int J Mol Sci. 2019 Jan 17;20(2):390. doi: 10.3390/ijms20020390 (PMC6359015; doi:10.3390/ijms20020390)
Supplement: Supplementary file 1 [file ijms-20-00390-s001.zip › ijms-402100-supplementary-XML/ijms-402100-supplementary Figures S1-S4.pdf]

Supplementary Figure S1

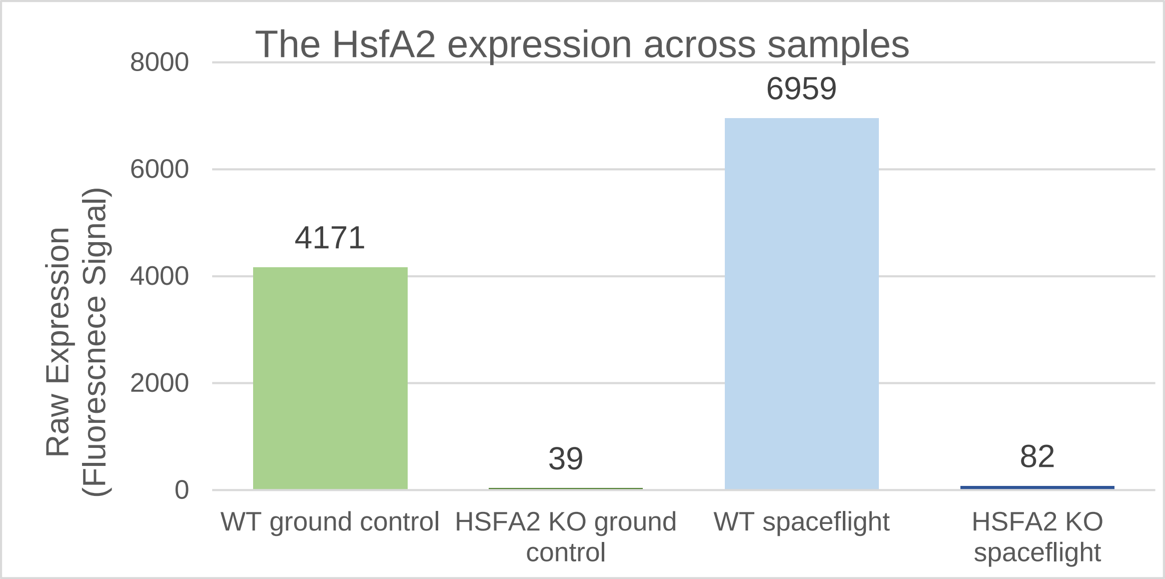

Supplementary Figure S1. Bar chart showing the HsfA2 gene expression across samples.
The MAS5 normalized raw expression values of the HsfA2 transcript obtained from the microarray fluorescent signal were plotted for the ground control samples (shades of green) and spaceflight samples (shades of blue) and for WT (lighter tone) and HSFA2 KO cells (darker tone).

Supplementary Figure S2

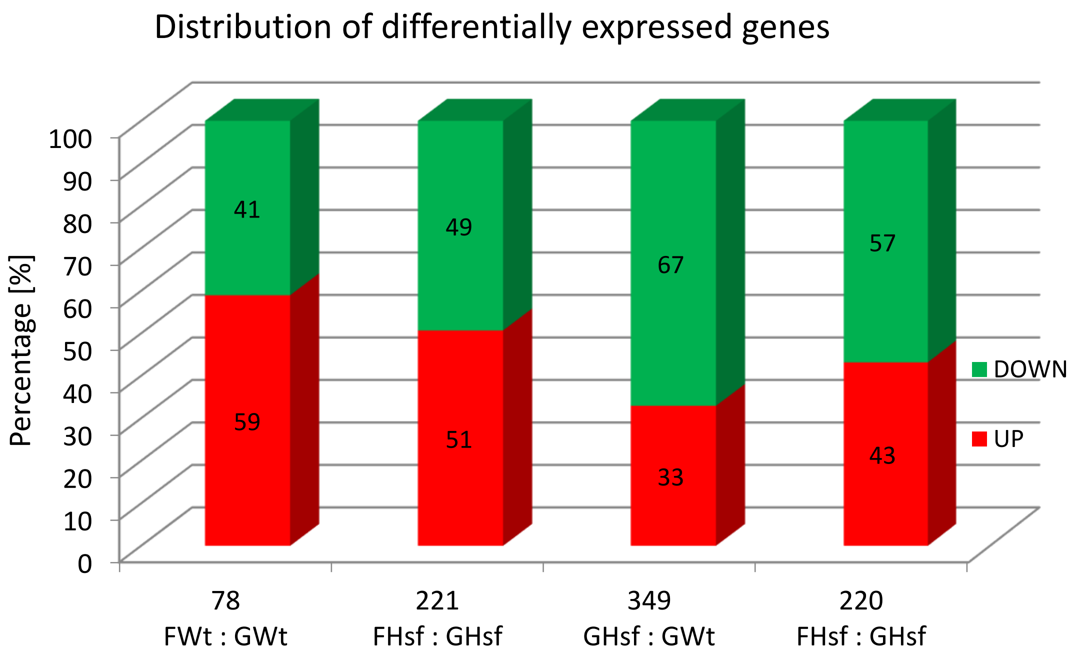

Supplementary Figure S2. Bar chart of the percentage distribution of the significantly differentially upregulated and downregulated genes within each comparison group. The total number of significantly differentially expressed genes was set as 100%.

Supplementary Figure S4 - The distribution of selected gene ontology (GO) terms

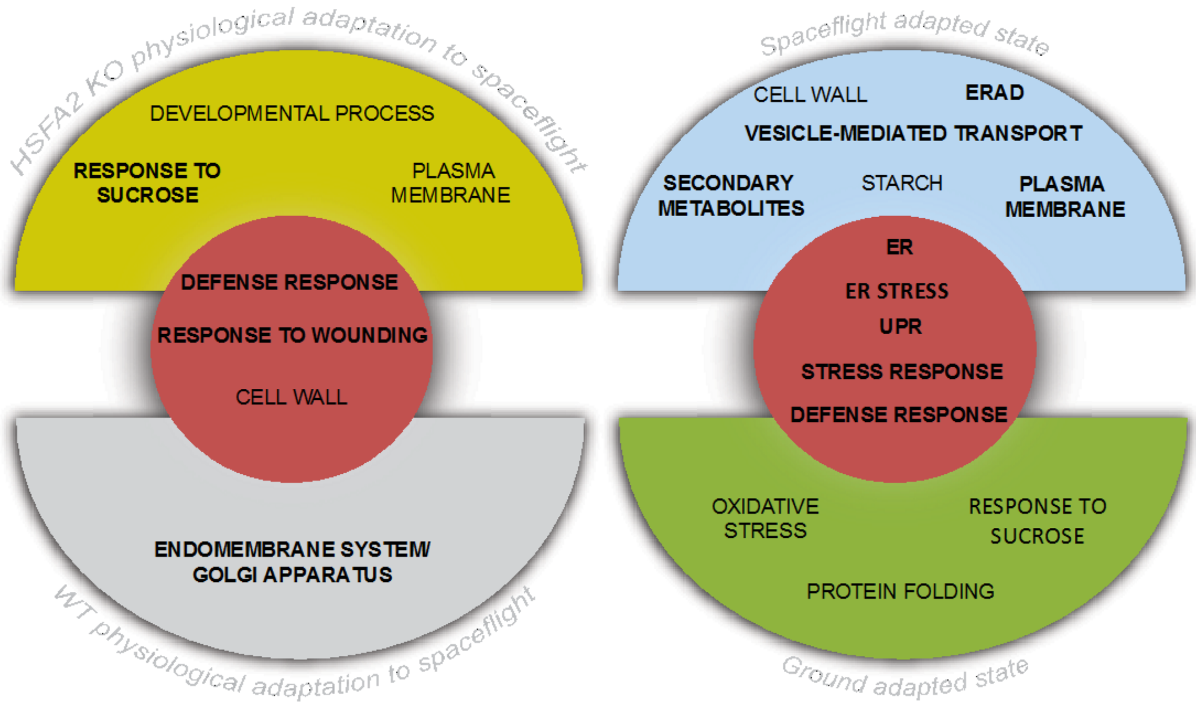

Figure S4: Graphical presentation of the distribution of selected gene ontology (GO) terms assigned to the significantly differentially expressed genes in four comparison groups:
LEFT bottom half-circle: FWt : GWt comparison group;
LEFT upper half-circle: FHsf : GHsf comparison group;
LEFT connecting circle in the middle: GO terms represented by genes of both spaceflight to ground control (F : Gs) comparison groups.
RIGHT bottom half-circle: GHsf : GWt comparison group;
RIGHT upper half circle: FHsf : FWt comparison group;
RIGHT connecting circle in the middle: GO terms represented by genes of both HSFA2 KO to WT cells (Hsf : Wt's) comparison groups.
The text in bold represents the processes associated with the upregulated genes, the normal text represents the processes associated with the downregulated genes.

Supplementary Figure S3 - AgriGO Parametric Analysis of Gene set Enrichment (PAGE)

GO Information					CM		GHsf : GWt			FHsf : FWt		
	GO Term	Ont	No	Description	1	2	Z-score	Mean	FDRnbsp;	Z-score	Mean	FDRnbsp;
1	GO:0051704	P	11	multi-organism process			1.8	0.5	0.067	0.17	-0.15	0.86
2	GO:0016043	P	23	**cellular component organization**			-2.1	-1.1	0.033	-2.3	-0.93	0.019
3	GO:0003723	F	17	RNA binding			1.9	0.37	0.052	0.076	-0.2	0.94
4	GO:0017111	F	20	nucleoside-triphosphatase activity			1.7	0.23	0.088	-1.1	-0.58	0.26
5	GO:0016818	F	20	hydrolase activity, acting on acid anhydrides, in phosphorus-containing			1.7	0.23	0.088	-1.1	-0.58	0.26
6	GO:0016817	F	20	hydrolase activity, acting on acid anhydrides			1.7	0.23	0.088	-1.1	-0.58	0.26
7	GO:0016462	F	20	pyrophosphatase activity			1.7	0.23	0.088	-1.1	-0.58	0.26
8	GO:0005783	C	12	**endoplasmic reticulum**			1.8	0.44	0.075	-0.0049	-0.23	1
9	GO:0019748	P	19	**secondary metabolic process**			1.5	0.15	0.14	2.6	0.63	0.0095
10	GO:0006350	P	27	transcription			0.51	-0.23	0.61	1.7	0.24	0.096
11	GO:0048856	P	21	anatomical structure development			1.1	-0.0024	0.28	-1.8	-0.8	0.069
12	GO:0000003	P	15	reproduction			0.19	-0.31	0.85	-2.1	-0.99	0.039
13	GO:0009791	P	12	post-embryonic development			-0.3	-0.52	0.77	-2.1	-1.1	0.035
14	GO:0048608	P	10	reproductive structure development			0.22	-0.27	0.83	-2.2	-1.2	0.025
15	GO:0003006	P	10	reproductive developmental process			0.22	-0.27	0.83	-2.2	-1.2	0.025
16	GO:0022414	P	14	reproductive process			0.62	-0.12	0.54	-2.3	-1.1	0.019
17	GO:0005886	C	52	**plasma membrane**			1.3	-0.11	0.21	2	0.17	0.045

Uniquely or commonly affected gene function in ground adapted states and spaceflight adapted states of the two genotypes as seen with the AgriGO Parametric Analysis of Gene set Enrichment (PAGE) tool (http://bioinfo.cau.edu.cn/agriGO). Functional category comparisons among the differentially expressed genes in the two homeostatic transcriptome comparisons using Parametric Analysis of Gene Expression. Yellow-to-red and cyan-to-blue color scale in CM (color mode) indicate whether the representation of the genes encompassed in that term is up- or down-regulated as a group. The Gene Groups comprising each analysis are provided in the first column. No (number) represents the number of genes included in each GO term. Onto refers to the Ontology group represented (P-biological process, F-molecular function, C-cellular component). The CM comparison categories are 1- G^{Hsf} : G^{Wt} and 2-F^{Hsf} : F^{Wt} , and the numerical values supporting the color rendering are provided to the right of the CM columns. FDR refers to false discovery rates.

The (PAGE) tool was used to evaluate specifically enriched biological processes of the 349 genes differentially expressed in the ground adapted state (G^{Hsf} : G^{Wt}) and 221 genes in the spaceflight adapted state (F^{Hsf} : F^{Wt}). There was one GO term represented by both gene sets, **a cellular compartment organization** (including genes associated with endoplasmic reticulum: At4g02980- ABP1 endoplasmic reticulum auxin binding protein 1, At1g08220; cell wall: At2g06850- XTH4 xyloglucan endotransglucosylase/hydrolase 4, At4g28250- EXPB3 expansin B3, At3g19770- VPS9A Vacuolar sorting protein 9 (VPS9) domain; with nucleosome assembly and chromatin remodeling: At1g07790- HTB Histone superfamily protein, At3g09480- Histone superfamily protein, At1g48620- HON5 high mobility group A5, At3g18035- HON4 winged-helix DNA-binding transcription factor family protein, At1g21700- SWI3C SWITCH/sucrose nonfermenting 3C, At1g14410- WHY1 ssDNA-binding transcriptional regulator, At5g48600- SMC3 structural maintenance of chromosome 3) (see Figure S3 in the Supplementary Material).

The **endoplasmic reticulum** GO term (including At5g45420- maMYB Duplicated homeodomain-like superfamily protein, At2g15090- KCS8 3-ketoacyl-CoA synthase 8, At2g47470- UNE5 alias ATPDIL2-1 PDI-LIKE 2-1 thioredoxin family protein, At1g72280- ERO1 oxidoreductins 1, At4g02980- ABP1 endoplasmic reticulum auxin binding protein 1) was represented by the genes of the ground adapted state differentially expressed between the WT and HSAF2 KO cells. The **secondary metabolic process** Go term (glucosinolate biosynthetic process At4g03070- AOP1.1 2-oxoglutarate (2OG) and Fe(II)-dependent oxygenase superfamily protein; nitrile biosynthetic process At5g48180- NSP5 nitrile specifier protein 5; flavonoid biosynthetic process At1g17020 SRG1 senescence-related gene 1; camalexin biosynthetic process At2g38470- WRKY33 WRKY DNA-binding protein 33), and **plasma membrane** GO term (plasma membrane transporter At3g15380- Plasma-membrane choline transporter family protein; plasma membrane receptors: At5g65240- Leucine-rich repeat protein kinase family protein, At5g48380- BIR1 BAK1-interacting receptor-like kinase 1, At5g25930- Protein kinase family protein with leucine-rich repeat domain; plasma membrane lipid metabolic process: At1g54010- GDSL-like Lipase/Acylhydrolase superfamily protein; plasma membrane associated with cell wall biosynthesis: At5g15630- IRX6 COBRA-like extracellular glycosyl-phosphatidyl inositol-anchored protein family, At2g02100- PDF2.2 low-molecular-weight cysteine-rich 69) were represented by the genes of the spaceflight adapted state differentially expressed between the WT and HSAF2 KO cells.
